# Immune modulation of liver sinusoidal endothelial cells by melittin nanoparticles suppresses liver metastasis

**DOI:** 10.1038/s41467-019-08538-x

**Published:** 2019-02-04

**Authors:** Xiang Yu, Lu Chen, Jianqiao Liu, Bolei Dai, Guoqiang Xu, Guanxin Shen, Qingming Luo, Zhihong Zhang

**Affiliations:** 1 Britton Chance Center for Biomedical Photonics, Wuhan National Laboratory for Optoelectronics-Huazhong University of Science and Technology, Wuhan, Hubei 430074 China; 2 MoE Key Laboratory for Biomedical Photonics, Collaborative Innovation Center for Biomedical Engineering, School of Engineering Sciences, Huazhong University of Science and Technology, Wuhan, Hubei 430074 China; 3 Department of Immunology, Tongji Medical College, Huazhong University of Science and Technology, Wuhan, Hubei 430030 China

## Abstract

Liver sinusoidal endothelial cells (LSECs) are responsible for the immunologic tolerance of liver which is a common site for visceral metastases, suggesting its potential role as an target for cancer immunotherapy. However, targeted modulation of LSECs is still not achieved thus far. Here, we report LSECs are specifically targeted and modulated by melittin nanoparticles (α-melittin-NPs). Intravital imaging shows that LSECs fluoresce within 20 s after intravenous injection of α-melittin-NPs. α-melittin-NPs trigger the activation of LSECs and lead to dramatic changes of cytokine/chemokine milieu in the liver, which switches the hepatic immunologic environment to the activated state. As a result, α-melittin-NPs resist the formation of metastatic lesions with high efficiency. More strikingly, the survival rate reaches 80% in the spontaneous liver metastatic tumor model. Our research provides support for the use of α-melittin-NPs to break LSEC-mediated immunologic tolerance, which opens an avenue to control liver metastasis through the immunomodulation of LSECs.

## Abstract (web summary)

Liver sinusoidal endothelial cells are known to promote immune tolerance in liver. Here, the authors target these cells using melittin nanoparticles and show alterations in the liver immune environment and suppression of liver metastases.

## Introduction

Metastasis is responsible for as much as 90% of cancer-associated mortality^1^. The liver is a distant metastasis site that is often involved in many gastrointestinal cancers, particularly colorectal cancer, and extragastrointestinal cancers, including breast cancer and melanoma. In the currently approved treatment regimen, surgical resection represents the only potentially curative treatment for resectable liver metastasis. However, over one-half of those patients still develop recurrent liver metastases within 2 years and the 5-year survival is about 20–50%^2,3^. Immunotherapy, such as immune checkpoint inhibitors^4^, chimeric antigen receptor cell therapies^5^ and tumor-associated antigen cancer vaccines^6^, is the most promising therapeutic strategy for cancer; however, it is often unsatisfactory for preventing liver metastasis. In fact, the liver is a unique immunological organ with strong intrinsic immune suppression environment, which contributes to the development of liver metastasis and impedes the effect of immunotherapeutic interventions in the tumor environment^7,8^.

Recently, some strategies aimed to overcome the inherent tolerogenicity of liver, including reducing suppressor lymphocyte (e.g., Tregs, MDSCs) and activating hepatic effector cells (e.g., NK, T cells) in the liver, thereby increasing the potential to resist liver metastasis. For example, the engineered CXCL12 trap achieves liver-specific targeting of CXCL12 and reduces the occurrence of liver metastasis by inhibiting the recruitment of CXCR4^+^ immunosuppressive cells^9^. Entolimod, a Toll-like receptor 5 agonist, also suppresses liver metastasis by increasing the recruitment and activation of NK cells^10^. However, these strategies do not specifically affect liver-resident immunocytes, especially antigen presenting cells (APCs). Modulation of the tolerogenic APCs in the liver should be a potent strategy to activate the specific anti-tumor immune response and eliminate tumor metastasis^7^. Liver sinusoidal endothelial cells (LSECs), which comprise ~50% of the non-parenchymal cells in the liver and form the fenestrated wall of the hepatic sinusoids, have the potential to act as APCs^11,12^. Usually, LSECs play an important role in the inherent tolerogenicity of the liver, mainly due to the low levels of expression of costimulatory molecules and their ability to produce IL-10 and TGF-β^7,13^. This means that LSECs fail to function as professional APCs and do not drive CD4^+^ T cells into differentiating into Th1 cells^14^. Moreover, the unique tolerogenic phenotype of B7-HI^high^ CD80/CD86^low^ on the surface of LSECs results in the imbalance of stimulatory and inhibitory signals, leading to CD8^+^ T-cell tolerance^15,16^. In addition, LSECs could influence the dendritic cell (DC) costimulatory function to indirectly regulate the functional states of CD4^+^ and CD8^+^ T cells^17^. As versatile non-migratory APCs in the liver, LSECs do not require the time-consuming steps involved in APC migration to lymphatic tissue, and activated LSECs could mediate the recruitment of immune cells to the liver^18^. Thus, LSECs have the potential to serve as immunotherapy target, and the selective activation of LSECs to break their tolerance-inducing properties has the capacity to awake anti-tumor response in liver. However, it is very challenging to target and modulate LSECs specifically due to the many phagocytic cell subpopulations in the liver and the lack-of-specific phagocytic receptors on LSECs.

Cationic host defense peptides are multifunctional peptides of fewer than 100 amino acids that are evolutionarily conserved molecules in the innate immune system and that display a wide range of immunomodulatory activities, including modulating the pro-inflammatory response, enhancing chemoattraction, promoting cellular differentiation, activating the innate and adaptive compartments, and modulating autophagy^19–22^. As one of the natural cationic host defense peptides, melittin has 26 amino acid residues (GIGAVLKVLTTGLPALISWIKRKRQQ) and possesses multiple biological effects, including tumor cell cytotoxicity and immunomodulatory effects^23^. It has also been reported that peptides containing the RXR or RXXR sequences have the ability to target LSECs^24^. Thus, we speculate that melittin should have the potential ability to target and modulate LSEC. However, melittin itself cannot be used to affect LSECs in vivo due to its main side effect, hemolysis^25^. Previously, we developed a 20-nm core-shell peptide-lipid nanoparticle (α-peptide-NP) that is precisely controlled by an amphipathic α-helical peptide (DWFKAFYDKVAEKFKEAF-NH_2_)^26^. Subsequently, we designed a hybrid peptide based on the α-helical peptide and the cytolytic melittin peptide, which had a strong α-helical configuration and interacted with phospholipids to form a self-assembled lipid nanoparticle, denoted as α-melittin-NP. The lipid layer of the α-melittin-NP shields the toxicity of melittin or α-melittin, making it possible to administer melittin via intravenous injection while retaining the melittin-induced toxicity in tumor cells^27^. Given that the melittin peptide contains the RKR sequence and possesses immunomodulatory effect, we hypothesize that the α-melittin-NPs target and modulate LSECs to become an activated APCs, changing the hepatic environment from its immune tolerant state to activated state; in addition, α-melittin-NPs may be inclined to execute the cytotoxic effects in tumor cells to release tumor-associated antigens^27^. In this study, we monitor the dynamics uptake of α-melittin-NPs in the hepatic sinusoid using real-time intravital imaging and detect the targeting ability and stimulating effect of α-melittin-NPs using flow cytometry (FCM) and transcriptome RNA-seq analysis. We demonstrate the expected immunomodulatory effect of α-melittin-NPs on LSECs and observe that the intravenous administration of α-melittin-NPs successfully blocks metastases formation and clearly prolongs the survival rates in multiple experimental liver metastasis models as well as in the spontaneous liver metastatic model of breast cancer.

## Results

### α-melittin-NPs quickly target to LSECs **in vivo**

**Fig. 1:**
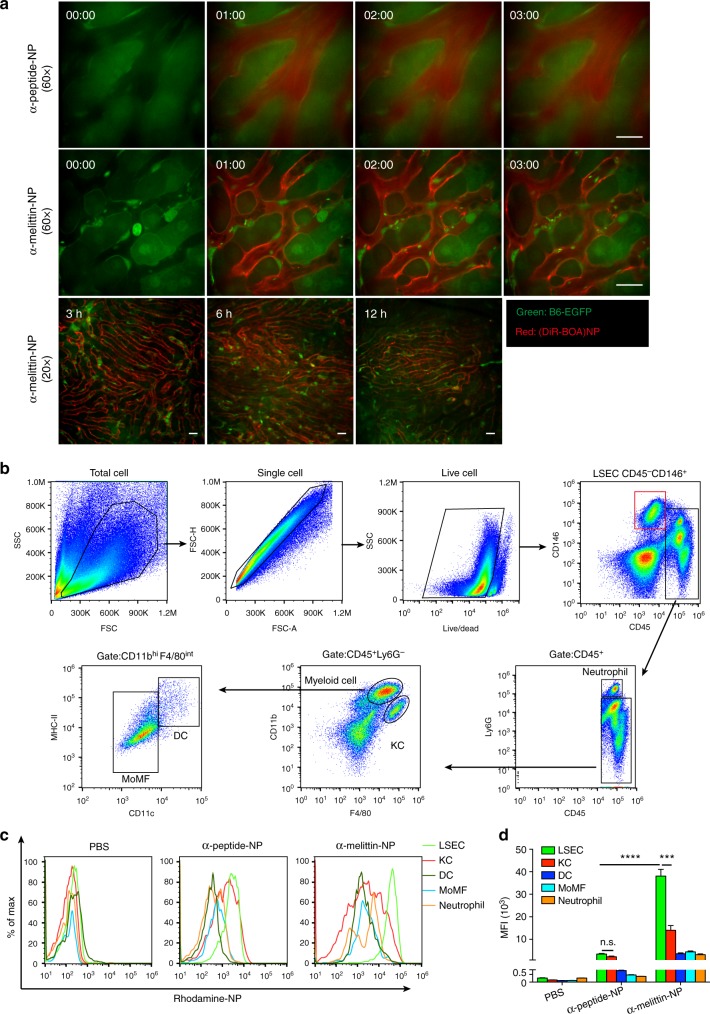
α-melittin-NPs target the LSECs in vivo. **a** Intravital imaging of α-melittin-NPs (middle and lower panels) and α-peptide-NP control (upper panel) in the liver. α-melittin-NPs and α-peptide-NPs were labeled with DiR-BOA (red), a lipid-anchored near-infrared fluorophore. The Actb-EGFP mice were used to visualize the structure of the liver. Time is indicated as min:sec in the upper and middle panels. Images are representative of three trials. Scale bar, 20 μm. **b** Gating strategies to distinguish the hepatic myeloid cells. The cells were pre-gated on single cells and live cells. Live cells were pre-gated on CD45 and CD146. Liver sinusoidal endothelial cells were defined as CD45^–^CD146^+^ cell and leukocytes as CD45^+^ cells. In the leukocytes gate, the cells were pre-gated on Ly6G^–^. KCs were defined as CD45^+^CD11b^int^F4/80^hi^ cells, and myeloid cells were gated as CD11b^hi^F4/80^int^ cells and further divided into DCs (CD11c^hi^MHC-II^hi^) and MoMFs (CD11c^int^MHC-II^low^). **c** Representative histograms indicating mean fluorescence intensity (MFI) of Rhodamine-NP. **d** Quantitative data of the MFI of Rhodamine-NPs in cell populations (*n* = 4 mice per group). Error bars indicate SEM. n.s. not significant; ****P < 0.0001; ***P < 0.001; by one-way ANOVA followed by Bonferroni’s post hoc test

To demonstrate our hypothesis of α-melittin-NP targeting LSECs, α-melittin-NPs core-loaded with DiR-BOA, a lipid-anchored near-infrared fluorophore, were used to monitor their distribution in liver. In addition, we used Actb-EGFP mice, in which EGFP is expressed uniformly in all cells except the erythrocytes and hair, to display the structure of the hepatic lobule and the cells in liver sinusoid via intravital imaging. The time-lapse microscopy imaging showed that LSECs fluoresced within 20 s after intravenous injection, and the boundaries of hepatic sinusoid were clearly visible (Fig. 1a and Supplementary Movie 1). The wild-field imaging of organs confirmed that α-melittin-NPs mainly accumulated in the liver rather than in the spleen, kidney or lung (Supplementary Fig. 1). α-peptide-NPs core-loaded with DiR-BOA were used as control carrier without the peptide sequence of melittin and were observed flowing through the hepatic sinusoid and rarely labeling LSECs (Fig. 1a and Supplementary Movie 1). The long-term intravital imaging data revealed that α-melittin-NPs were mainly located at the wall of the hepatic sinusoid even 12 h after injection; whereas, the control nanoparticles were taken up by hepatic parenchymal cells and diffused throughout the liver (Supplementary Fig. 2). To further confirm that the strip-like distribution of α-melittin-NPs in the hepatic sinusoid was attributable to its specific targeting of LSECs, we used multicolor flow cytometry to analyze the uptake of α-melittin-NP by the non-parenchymal cells in the liver, such as LSECs, Kupffer cells (KCs), DCs, monocyte-derived macrophages (MoMFs), and neutrophils. Normally, LSECs are defined as CD45^–^CD146^+^ cells and leukocytes as CD45^+^ cells. In the leukocytes gate, the cells were pre-gated on Ly6G^–^. KCs were defined as CD45^+^CD11b^int^F4/80^hi^ cells, and myeloid cells were gated as CD11b^hi^F4/80^int^ cells and further divided into DCs (CD11c^hi^MHC-II^hi^) and MoMFs (CD11c^int^MHC-II^low^) (Fig. 1b). Figure 1c shows representative FCM histograms for the fluorescent intensity of nanoparticle within the cell subsets in the liver. The quantificational data of the mean fluorescence intensity (MFI) show that the ability to uptake α-melittin-NPs is highest in LSECs, compared to KCs (2.7-fold), DCs (10.6-fold), MoMFs (8.6-fold), and neutrophils (11-fold) (Fig. 1c, d). LESCs also displayed non-specifically phagocytic abilities, similar to those of KCs, which took up a certain number of α-peptide-NPs. Due to the melittin peptide sequence in α-melittin-NP, LSECs are more efficient at uptaking α-melittin-NPs than the α-peptide-NP control, with a 11-fold difference (Fig. 1d). Thus, these data indicated that α-melittin-NPs mainly target the LSECs in vivo via melittin peptide sequence.

### Immunomodulatory function of α-melittin-NPs to LSECs

**Fig. 2:**
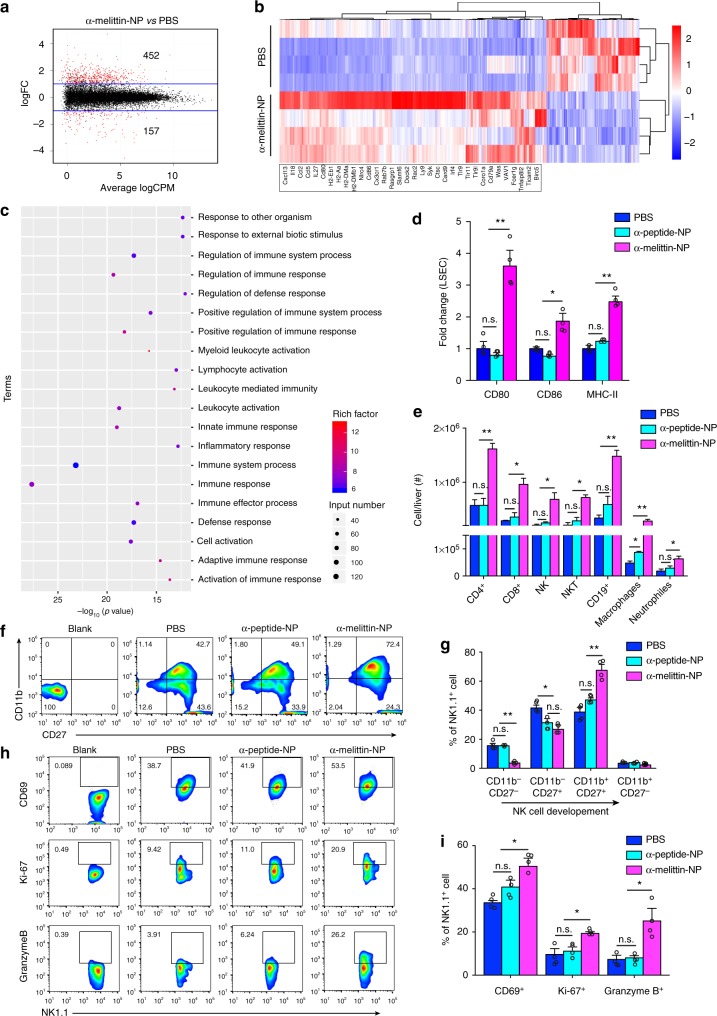
α-melittin-NPs modulate the activation of LSECs and the infiltration of leukocytes in the livers of normal mice. **a** The number of genes passing the *q* < 0.05 threshold and LogFC > 1 in the α-melittin-NP group (*n* = 4 mice per group). **b** Heatmap of the 452 upregulated and 157 downregulated genes in the α-melittin-NP-treated LSECs. Some representative genes related to immune response are showed below. **c** Scatter plot for GO enrichment results in the upregulated genes. The dot size indicates the number of DEGs contained in the GO terms, and the dot depth indicates the extent of rich factor enrichment. **d** The quantitative data of the MFIs of CD80, CD80, and MHC-II in the LSEC cell population after different treatments. **e** Absolute numbers of immune cell subsets in the whole liver. **f**, **g** Representative FCM plots **f** and percentages **g** of CD3^–^NK1.1^+^ cells divided based on their expression of CD11b and CD27. **h**, **i** Representative FCM plots **h** and percentages **i** of NK cells according the expression of CD69, Ki-67, and granzyme B. Error bars indicate SEM. n.s. not significant; **P < 0.01; *P < 0.05; by one-way ANOVA followed by Bonferroni’s post hoc test

Having confirmed the specific targeting of α-melittin-NP to LSECs, we were interested in exploring the ability of α-melittin-NP to immunomodulate LSECs. First, we isolated LSECs with CD146 immunomagnetic sorting followed by transcriptome RNA-seq analysis. Compared to the PBS group, 609 transcripts at a fold change >1 and false discovery rate (FDR) <0.05 were differentially expressed, among which 452 genes were upregulated and 157 genes were downregulated (Fig. 2a) according to the fragments per kilobase of exon per million mapped reads (FRKM) values. As highlighted on the heatmap, the upregulated genes, unlike the downregulated genes, were mainly involved in immune responses (Fig. 2b and Supplementary Fig. 3). Then, we analyzed the obtained differentially expressed genes (DEGs) using Gene Ontology (GO) enrichment analysis. The most enriched GO terms are summarized in Fig. 2c. We found a series of terms related to the immune system, such as immune response, leukocyte activation, innate immune response, and regulation of immune response. To identify the involvement of the canonical pathways in immune response, the DEGs were analyzed using the Kyoto encyclopedia of genes and genomes (KEGG) database. The results revealed that 30 pathways had at least one DEG, among which the most significantly enriched pathways (*P* < 0.05) were the natural killer cell-mediated cytotoxic, chemoking signaling pathway, antigen processing and presentation, and leukocyte transendothelial migration. These results indicated that α-melittin-NPs possess vigorously immunostimulatory properties that affect the LSECs at the level of transcription. Next, according to the transcriptomic information, we verified the immune stimulation by α-melittin-NP at the protein level. The expression of costimulatory molecules on the LSECs was detected using flow cytometry, and the production of cytokines/chemokines in the liver was measured by LEGENDplex^TM^ mouse inflammation and chemokine panel array. The data showed that compared to the LSECs in the control mice, LSECs in the α-melittin-NP-treated mice increased the MFI values for costimulatory molecules, with 4.5-fold increase in CD80, 1.6-fold increase in CD86, and twofold increase in MHC-II (Fig. 2d). However, there were no changes of the expression of costimulatory molecules on other phagocytic cell subpopulations (Supplementary Fig. 4). By analyzing the data from the multiplexed cytokine/chemokine array, we found that the expression levels of cytokines and chemokines involved in leukocyte activation and migration, such as IL-1α, CXCL9 (MIG), CXCL10 (IP-10), CXCL13 (BLC), CCL3 (MIP-1α), CXCL1 (KC), CCL4 (MIP-1β), and CCL5 (RANTES), were significantly increased in the α-melittin-NP-treated mice compared to the levels in the control mice (Supplementary Fig. 5). Considering the risk of possible liver damage induced by high cytokine/chemokine levels, we collected the blood samples after the administration of a single dose and multiple doses to evaluate the impact on liver function. Between the α-melittin-NP and control groups (PBS and α-peptide-NP), the biochemical analysis results showed no significant differences in the levels of hepatic function parameters [e.g., albumin (Alb), total bilirubin (T-Bil), and aspartate aminotransferase (AST)] at 24 h after the administration of a single dose or multiple doses (Supplementary Fig. 6b–d). The only difference observed was that the alanine aminotransferase (ALT) level was slightly elevated compared to that of the control groups, but was still within the normal range (27–195 IU/L, C57BL/6 mice, female, 8–10 weeks) at 24 h after the administration of multiple doses. In addition, we detected these parameters at 48 h after the administration of multiple doses and found that the α-melittin-NP group did not have an increased ALT level (Supplementary Fig. 6e), indicating that the impact of the α-melittin-NPs on the ALT level was transient. Therefore, we confirmed that the intravenous administration of α-melittin-NP efficiently elicited LSEC activation and reversed the immune microenvironment with dramatic changes in the cytokine/chemokine milieu in the liver.

In addition, we attempted verify the immunomodulatory function of α-melittin-NPs in vitro because of the complexity of the in vivo liver microenvironment. The isolated primary LSECs were cultured without treatment or were stimulated with α-melittin-NPs for 3 or 24 h. The exposure of the LSECs to α-melittin-NPs at 5 μM increased the expression of costimulatory molecules (CD80 and MHC-II), whereas there was no difference between the lower concentration group (2.5 μM) and the control groups (medium and α-peptide-NPs) (Supplementary Fig. 7a). Additionally, the treatment with 5 μM α-melittin-NPs significantly upregulated the mRNA levels of cytokines (IL-1α, IL-2, IL-12, and IL-18) and chemokines (CXCL2, CXCL9, CXCL10, CXCL13, and CCL3**–**5) compared to the other three groups (Supplementary Fig. 7b). We also showed that the application of α-melittin-NPs at 5 μM induced the increased expression of selected cytokines/chemokines at the protein level in vitro (Supplementary Fig. 7c). Thus, these results indicated that the α-melittin-NPs mediate the activation of LSECs in a concentration-dependent manner.

### Characteristic of switched microenvironment in the liver

Motivated by the changes in gene expression in LSECs and the protein levels in the liver after targeting with α-melittin-NP, we analyzed the cell types and absolute numbers of infiltrating leukocytes in the liver by flow cytometry. The data showed that the absolute numbers of innate immune components, including NK cells, natural killer T (NKT) cells, macrophages and neutrophils, and the adaptive components, including B cells and CD4^+^ and CD8^+^ T cells, increased in the α-melittin-NP group compared to the control group (Fig. 2e). However, there were no significant differences between the mice treated with or without α-melittin-NPs in terms of the percentages of these cell subpopulations (Supplementary Fig. 8a–e). Given the critical tumoricidal role of NK cells in the innate immune response and the elevated level of IL-18 and IL-1α, which have been reported as mediating NK cell maturation and tumoricidal activity^28,29^, we sought to determine whether α-melittin-NP treatment stimulates the differentiation and maturation of hepatic NK cells (gated by NK1.1^+^ and CD3^–^). Usually, NK cell development is a four-stage process that starts with a CD11b^–^CD27^−^ stage and proceeds through the following stages: CD11b^–^CD27^+^→CD11b^+^CD27^+^→CD11b^+^CD27^–^, as previously reported^30^. This developmental program is associated with the progressive acquisition of NK-cell effector functions. FCM staining of hepatic NK cells showed that compared with the control treatments, α-melittin-NP treatment significantly reduced the frequencies of less developed NK-cell populations (CD11b^–^CD27^–^ and CD11b^–^CD27^+^) and increased the frequencies of developed NK cell populations (CD11b^+^CD27^+^) (Fig. 2f, g). In addition, compared with the PBS or α-peptide-NP control groups, α-melittin-NP treatment-induced higher percentages of CD69, Ki-67, and granzyme B, the markers of NK-cell activation, proliferation, and lytic activity, respectively (Fig. 2h, i). In addition to NK cells, we also analyzed the phenotypic changes in T cells. FCM analysis showed that α-melittin-NPs had no effect on the percentages of ICOS, Ki-67, granzyme B, and Tim3 of CD4^+^ and CD8^+^ T cells (Supplementary Fig. 8f–i). These results indicated that administration of α-melittin-NPs changes the hepatic immune state, with increased leukocyte infiltration and NK-cell maturation.

### α-melittin-NPs suppress experimental liver metastasis

**Fig. 3:**
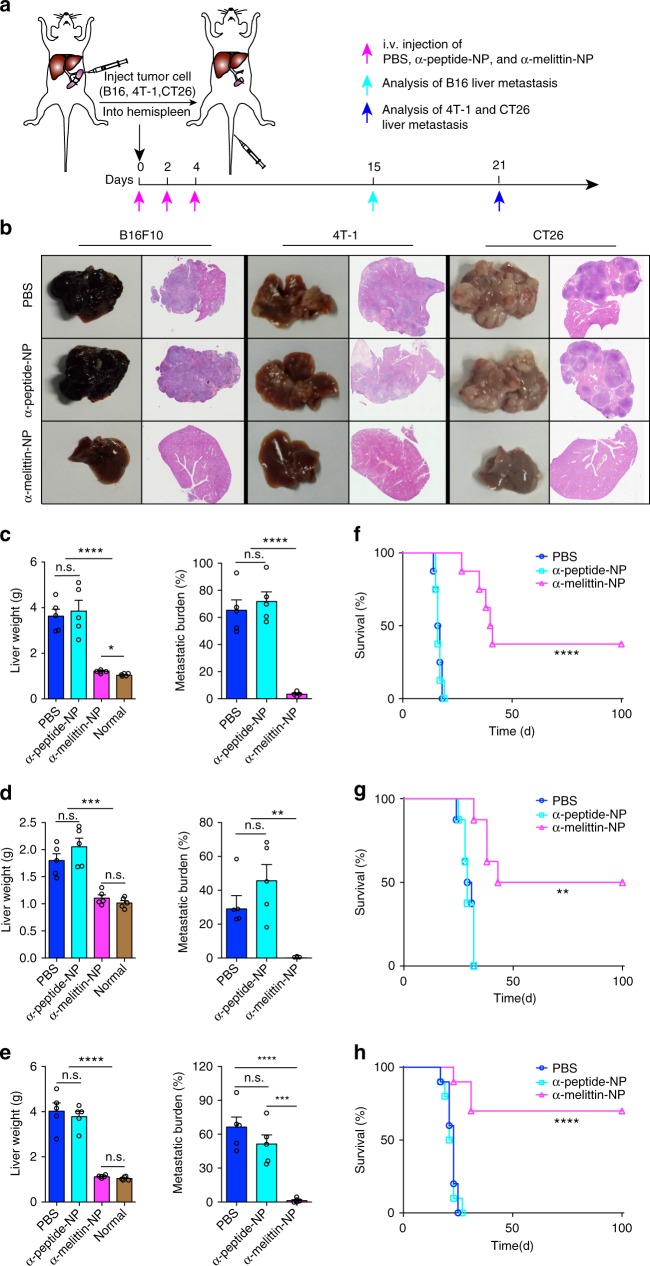
α-melittin-NPs suppress experimental liver metastasis and prolong survival in multiple tumor models. **a** The schematic of the hemisplenectomy and the timeline of treatment. The black arrow indicates the time point of the injection of tumor cells; the magenta arrows indicate the treatment time points; the cyan arrow indicates the end point of analysis of B16 liver metastasis; the blue arrow indicates the end points of analysis of 4T-1 or CT26 liver metastasis. **b** Representative pictures of the liver and hematoxylin and eosin (H&E) staining of liver sections from mice subjected to different treatments that were killed on day 15 (B16F10) or 21 (4T-1, CT26) after tumor intrasplenic injection. All individuals are shown in Supplementary Fig. 10a. **c**–**e** Quantification of liver weight (left panel) and metastatic burden (right panel) of B16F10 **c**, 4T-1 **d**, and CT26 **e** tumor models (*n* = 5 mice per group). Mice of same sex and age as in the experimental group were used as normal controls. **f**–**h** Survival rate for mice injected with B16F10 **f**, 4T-1 **g**, and CT26 **h**. In **f**, *n* = 8 C57BL/6 mice per group. In **g**, *n* = 8 BALB/c mice per group. In **h**, *n* = 10 BALB/c mice per group. Error bars indicate SEM. n.s. not significant; ****P < 0.0001; ***P < 0.001; **P < 0.01; *P < 0.05; by one-way ANOVA followed by Bonferroni’s post hoc test **c**–**e** or by log-rank Mantel-Cox test **f**–**h**

Having demonstrated α-melittin-NP-induced activation of LSECs and recruitment of immune cells, especially the maturation of NK cell, we speculated that the α-melittin-NP-modulated hepatic microenvironment change might resist the formation of hepatic metastasis. We tested this speculation using three mouse models of experimental liver metastasis, including melanoma (B16F10), triple-negative breast carcinoma (4T-1), and colon carcinoma (CT26). Inoculation was performed with 2 × 10^5^ (100 μl) of B16F10, 4T-1, and CT26 cells into the mouse hemispleens, which had been tied off and separated into two halves before the tumor injection. The half of the spleen that received the cells was resected 7 min after inoculation to decrease primary tumor growth in the spleen (Fig. 3a). In a series of experiments, treatment began ~3 h after tumor inoculation. The rapid migration of tumor cells to the liver often occurs within 5 min of inoculation^31^. Intravital imaging data confirmed that the tumor cells reached the liver and were detained in the hepatic sinusoid before treatment (Supplementary Fig. 9). After three times of treatment, we evaluated the effect of α-melittin-NPs on liver metastasis by measuring the weight of each liver and quantified the metastatic burden on hematoxylin and eosin (H&E) stained liver tissue sections in the three tumor models. The dissected livers were shown in Supplementary Figure 10a. The data showed that the mice that received PBS or α-peptide-NPs had heavier livers and higher metastatic burdens compared to those that received α-melittin-NP therapy (Fig. 3b–e). Interestingly, the liver weights of mice treated with α-melittin-NPs fluctuated within normal ranges and were not different than those of the normal control group in the 4T-1 and CT26 tumor models. In addition, the mice in control groups (PBS and α-peptide-NP) did show increases in body weight and elevations in the circulating amounts of AST and ALT, whereas the levels of those enzymes in the α-melittin-NP group remained within the normal ranges (Supplementary Fig. 10b,c). It is noteworthy that the level of AST in the control groups exceeded the instrument detection limits. Furthermore, to observe the survival rate, three additional batches of mice were injected with tumor cells and treated as shown in Fig. 3a, followed by observation for as long as 100 days. The mice were killed when one of the following conditions happened: drastic weight gain or loss greater than 10% of the total body weight within 1 week or visible signs of distress, such as dehydration, inactivity, or shortness of breath. The mice bearing B16F10, 4T-1, and CT26 tumors benefited greatly in terms of their survival rates after three times treatment, with 37.5%, 50%, and 70% survival, respectively, and were still alive 100 days after tumor inoculation (Fig. 3f–h). In contrast, mice in the PBS and α-peptide-NP groups of the three tumor models (B16F10, 4T-1, and CT26) were all dead by days 19, 33, and 27, respectively.

### α-melittin-NPs induce the generation of T-cell immunity

**Fig. 4:**
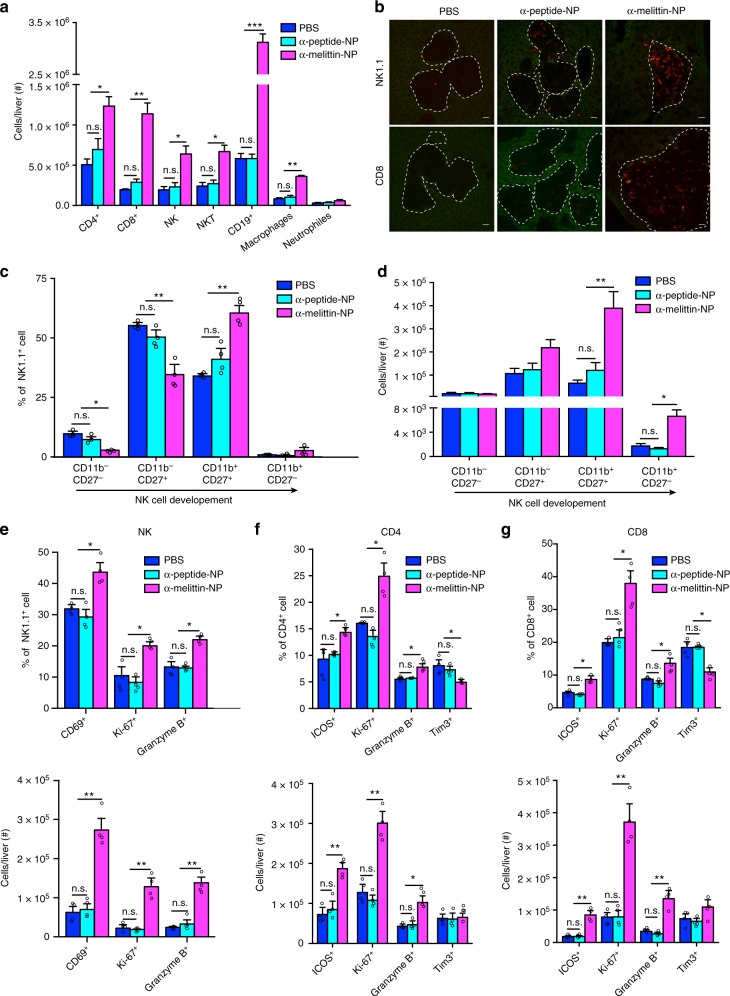
α-melittin-NPs induce the generation of T-cell immunity in the liver of tumor-bearing mice. Groups of mice (*n* = 4 mice per group) were injected intrasplenically with 2 × 10^5^ B16F10 cells in 100 μl of PBS and treated as shown in Fig. 3a. Hepatic lymphocytes were collected 6 days after injection by in vivo perfusion and in vitro digestion to assess the changes in leukocytes infiltration in the liver. Quantification was performed by flow cytometry. **a** Absolute numbers of innate and adaptive immune cells in the liver after different treatments. **b** Representative immunofluorescence images from the liver for NK1.1 (upper panel) and CD8 (lower panel). White dotted lines indicate metastatic lesions and red indicates the NK1.1^+^ or CD8^+^ cells. Scale bar, 20 μm. **c**, **d** The percentages **c** and absolute numbers **d** of NK cells according to their developmental phenotype. **e** The percentages (upper panel) and absolute numbers (lower panel) of NK cells according the expression of CD69, Ki-67, and granzyme B. **f**, **g** The percentages (upper panel) and absolute numbers (lower panel) of CD4^+^ T cells **f** and CD8^+^ T cells **g** according their expression of ICOS, Ki-67, granzyme B, and Tim3. Error bars indicate SEM. n.s. not significant; ***P < 0.001; **P < 0.01; *P < 0.05; by one-way ANOVA followed by Bonferroni’s post hoc test

To observe the changes in the tumor environment in the livers of tumor-bearing mice, we used the B16F10 tumor model and analyzed the cell types and absolute numbers of infiltrating leukocytes in the liver. The mice were injected with B16F10 tumor cells (2 × 10^5^) into the hemispleen and treated as shown in Fig. 3a. The lymphocytes were analyzed by immunofluorescence and flow cytometry on day 6 after administration of different treatments. As shown in Fig. 4a, the immunocyte infiltration in the α-melittin-NP group was characterized by increases in both innate immune components (NK cells, NKT cells, macrophages, and neutrophils) and adaptive components (B cells, CD4^+^, and CD8^+^ T cells). Immunofluorescence staining also revealed that the metastatic tumors in the α-melittin-NP-treated mice were highly infiltrated by both NK and CD8^+^ T cells. In contrast, the metastatic tumors in the PBS and α-peptide-NP groups had limited NK and CD8^+^ T-cell infiltration (Fig. 4b). Consistent with the immunomodulatory effect in normal mice, α-melittin-NP treatment resulted in increases in the percentages and numbers of differentiated and mature NK cells (Fig. 4c–e). However, α-melittin-NPs also induced the upregulations of ICOS, Ki-67, and granzyme B and the downregulation of Tim3 on the CD4^+^ and CD8^+^ T cells (Fig. 4f, g, upper panels). In addition, the absolute number of ICOS-positive, Ki-67-positive, and granzyme B-positive T cells increased after α-melittin-NP treatment, while there was no change in the absolute number of Tim3-positive T cells (Fig. 4f, g, lower panels).

Although α-melittin-NP could activate LSECs to support the local CD8^+^ T-cell response in the liver of tumor-bearing mice, the generation of systemic anti-tumor immune memory was not verified. Based on their distinct homing capacity and effector function, memory T lymphocytes contain distinct populations of effector memory (T_EM_) and central memory (T_CM_) cells. T_EM_ cells can migrate to inflamed peripheral tissues and display an immediate effector function, whereas T_CM_ cells can home to secondary lymphoid organs (e.g., spleen) and readily proliferate and differentiate to effector cells in response to antigenic stimulation^32^. Therefore, we measured the proportions of both effector memory cells (T_EM_) and central memory (T_CM_) cells at day 28 after treatment and used naive mice as a control group because the mice treated with PBS and α-peptide-NP were dead at that time. The data showed that the percentages of T_EM_ (CD62L^–^CD44^+^) and T_CM_ (CD62L^+^CD44^+^) cells in the blood, liver, and spleen were higher in the group of mice treated with α-melittin-NP (Supplementary Fig. 11). Taken together, these findings demonstrate that α-melittin-NPs not only stimulate the differentiation and maturation of NK cells, but also trigger T-cell-mediated systemic anti-tumor immune responses in the livers of tumor-bearing mice.

### Effect of α-melittin-NPs on the spontaneous liver metastasis

**Fig. 5:**
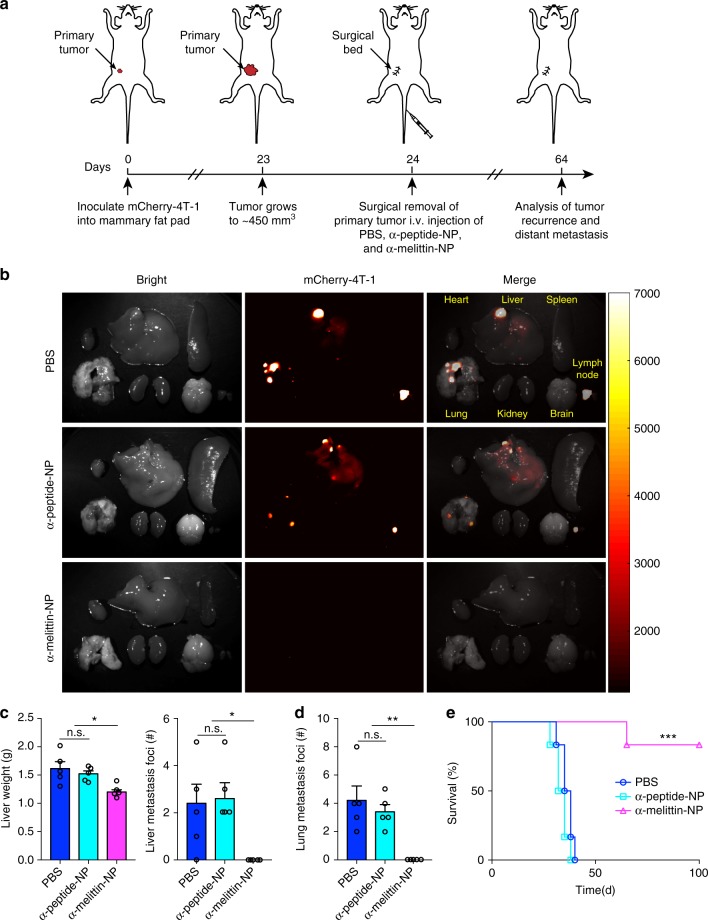
Effect of α-melittin-NPs in a spontaneous metastatic tumor model. **a** Schematic illustration of α-melittin-NP therapy in a mouse model of spontaneous liver metastasis. The mCherry-4T-1 cells (3 × 10^5^) were inoculated into the mammary fat pads of syngeneic BALB/c mice and the primary tumors were resected when they reached about 450 mm^3^ in volume following treatment with PBS, α-peptide-NPs or α-melittin-NPs on days 0, 7, and 14, respectively, post surgery. The numbers of metastatic lesions in different organs were quantified by whole-body fluorescence system. **b** Representative images of organ tumor burdens on day 64. **c** Quantification of the liver weights (left panel) and liver metastatic foci (right panel) of mice sacrificed on day 64 (*n* = 5 mice per group). **d** Quantification of the lung metastatic foci of mice sacrificed on day 64 (*n* = 5 mice per group). **e** Mouse survival was monitored over the course of 100 days post surgery (*n* = 6 mice per group). Error bars indicate SEM. n.s. not significant; ***P < 0.001; **P < 0.01; *P < 0.05; by one-way ANOVA followed by Bonferroni’s post hoc test **c**, **d** or by log-rank Mantel-Cox test **e**

Though the experimental metastasis model is highly reproducible and saves time when developing liver metastases, it hardly represents the natural metastatic process that involves tumor cell local invasion and extravasation into distant organs^33^. To assess the efficacy of α-melittin-NP treatment in a model that mimics a realistic process from the formation of the primary site to the development of liver metastasis, we prepared a spontaneous liver metastasis model. As shown in Fig. 5a, mCherry-expressing 4T-1 cells were inoculated into the mammary fat pads of syngeneic BALB/c mice, and the primary tumors were resected when they reached 450 mm^3^. The mice were divided into three treatment groups that received PBS, α-peptide-NP, and α-melittin-NP on days 0, 7, and 14, respectively, after the operation. On day 40 after the primary tumor resection, the livers and other organs were collected and analyzed using a wild-field fluorescent imaging system to observe tumor metastasis. Strong fluorescent signals of mCherry-4T-1 cells were clearly detected in the livers, lungs, and lymph nodes of mice treated with PBS or α-peptide-NPs, indicating the metastatic foci were easily generated in these organs (Fig. 5b). It is noteworthy that no detectable fluorescent signal of mCherry-4T-1 cells was observed in the organs of α-melittin-NP-treated mice. The quantitative data showed that the mice that received PBS or α-peptide-NPs had heavier livers and more liver and lung metastatic foci than did those that received α-melittin-NP therapy (Fig. 5c, d). Furthermore, the rates of primary tumor recurrence and distal organ metastasis (lung, lymph node) were almost 100% in the PBS and α-peptide-NP groups, with one out of five mice forming rare heart and kidney metastases in the PBS group, but the mice treated with α-melittin-NP rarely developed liver and lung metastases, with only one out of five mice experiencing primary recurrence and lymph node metastasis (Fig. 5d and Supplementary Table 1). We also prepared another batch of mice to observe the survival rate. Mice receiving α-melittin-NP therapy after surgery also benefited greatly in terms of their survival rate, with 80% of them still alive 100 days after the operation (Fig. 5e). In contrast, all mice in the PBS and α-peptide-NP control groups died within 40 days of the operation. These results indicate that α-melittin-NPs successfully suppress liver metastasis as well as metastasis to other organs in a spontaneous metastatic tumor model.

## Discussion

**Fig. 6:**
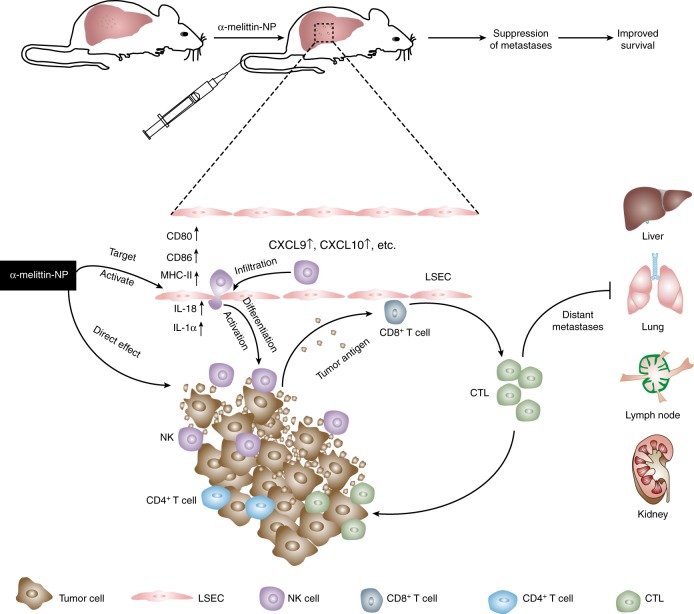
Schematic description of a plausible mechanism for α-melittin-NP-mediated suppression of liver metastasis. α-melittin-NPs specifically modulate LSECs, which involves the increased expression levels of costimulatory molecules (CD80, CD86, and MHC-II) and the release of cytokines/chemokines (IL-1α, IL-18, CXCL9, CXCL10, etc.). Once the LSECs have been activated by α-melittin-NPs, a variety of innate and adaptive immune cells migrate to the liver and generate protective T-cell immunity through coordination with NK cells to inhibit liver metastasis

The liver is a tolerogenic organ and is often associated with tumor metastasis from many primary sites. Immunotherapy appears to be more necessary for the liver than for other organs. However, no adequate experimental testing has been conducted to overcome immune suppression by modulating the intrinsically tolerogenic APCs in the liver. In this study, we demonstrate the ability of α-melittin-NP to reverse the tolerogenic liver environment by targeting and regulating the LSECs, thus resisting the development of liver metastasis from different tumor models. More strikingly, the survival rate reached 80% and was accompanied by the inhibition of metastasis to other organs in the spontaneous liver metastatic tumor model. The schematic diagram of α-melittin-NP tumor suppressive activity in the liver is shown in Fig. 6. The mechanisms were characterized by at least by the following aspects: (1) α-melittin-NP target and activate LSECs, thereby priming the adaptive anti-tumor immune response. LSECs have the potential to act as APCs and the activation of LSECs is a precondition for tumor antigen presentation and subsequent T-cell activation. In our study, we analyzed the obtained DEGs and confirmed that α-melittin-NPs induced the upregulation of CD80, CD86, and MHC class II genes (H2-Aa, H2-DMa, H2-DMb1, and H2-Eb1). In addition, we also found using flow cytometry analysis that the expression of CD80, CD86, and MHC-II increased on LSECs in vivo and in vitro. (2) α-melittin-NP-induced changes in immune infiltration and the activation of NK and T cells. Pre-existing tumor infiltrating lymphocytes (TILs) are a strong predictor of the response to immunotherapy. Our data show that α-melittin-NPs not only induced the increases in the numbers of innate immune cells and adaptive immune cells, but also promoted NK-cell maturation and T-cell activation in the livers of tumor-bearing mice. (3) α-melittin-NP had a direct cytotoxic effect on the metastatic tumors. Previously, we had demonstrated that α-melittin-NPs could induce B16F10 cell apoptosis and necrosis in vitro^27^. Thus, the direct effect of α-melittin-NPs on the metastatic tumor in the liver cannot be ignored.

The unique tolerogenicity of the liver is critical for preventing the induction of immunity against harmless antigens, such as gut-derived nutrients, antigens from aged or damaged normal cells and metabolic products^7^. Therefore, it is very important to maintain the balance between immune tolerance and immune activation. Our results showed that α-melittin-NPs did not increase the expression of costimulatory molecules on the KCs and liver DCs, which supported the induction of tolerance towards circulating and hepatocyte-derived antigens. At the same time, the activated LSECs had no effects on the activation of T cells in the normal mice (Supplementary Fig. 8f–i). Only when tumors existed in the liver did the activated LSECs significantly increase the activation, proliferation and lytic activity on CD4^+^ and CD8^+^ T cells (Fig. 4f, g). It seems that the balance of immunity status is tilted in favor of an anti-tumor response when there are tumor antigens in the liver. In addition to being an immunological organ, the liver is also a key metabolic organ and plays an important role in maintaining the homeostasis of body metabolism. We noted that the transcriptome RNA-seq analysis of LSECs indicated the most enriched GO terms in downregulated genes were mainly involved in metabolic process, such as the monocarboxylic acid metabolic process, the lipid metabolic process, the fatty acid metabolic process, and the steroid metabolic process (Supplementary Fig. 3). However, we found the liver displayed no significant changes in the metabolites levels, which was furtherly confirmed by measuring the mRNA expression levels of rate-limiting enzymes in glucose and lipid metabolic pathways at 24 h after α-melittin-NPs administration (Supplementary Fig. 12). In fact, overall liver metabolism is regarded as a function mainly of hepatocytes rather than LSECs because of the fact that the hepatocytes account for more than 90% of the total cellular volume of the liver^34^.

Although α-melittin-NPs consisted of three different components [1,2-dimyristoyl-sn-glycero-3-phosphocholine (DMPC), cholesterol oleate (CO), and the peptide)], the nanocarrier without the melittin peptide did not activate LSECs in vitro or in vivo and had no suppressive effects on liver metastasis in any of the three experimental liver metastasis models. Moreover, there were no differences in characteristics such as surface charge, particle size, and morphological properties between α-peptide-NPs and α-melittin-NPs^27,34^, suggesting that the melittin peptide rather than the structural characteristics of the nanoparticles played a direct role in the immunostimulatory effects of α-melittin-NPs. Previous reports on the immune-modulating effects of melittin were rather contradictory. Park et al. reported that melittin has inhibitory effects on the lipopolysaccharide (LPS)-induced expression of cyclooxygenase 2, cytosolic phospholipase A_2_, and inducible NO synthase by targeted inactivation of NF-κB^35^. In addition, Lee et al. reported that local administration of melittin significantly decreased the expression of various inflammatory cytokines in heat-killed *Propionibacterium acnes*-treated keratinocytes^36^. However, it was also reported that melittin could not block IL-1β-induced NF-κB activation, but rather significantly increased the mRNA levels of several pro-inflammatory genes^37^. Indeed, a recent report suggested that melittin triggered IL-1β and IL-18 release by the activation of the AIM2 inflammasome in the keratinocytes^38^. These paradoxical reports indicate that melittin itself is pro-inflammatory rather than anti-inflammatory in non-bacterial inflammatory processes. The data presented in our study corroborate the existence of immuno-modulating effects of melittin and its pro-inflammatory function. However, the pro-inflammatory function of melittin on LSECs may rely on the nanoparticle structure. In our previous study, we showed that the lipid layer of the nanoparticle shields red blood cells from the toxicity of melittin, making it possible to administer melittin via intravenous injection^27^. Thus, the nanoparticle structure of α-melittin-NPs provided precondition necessary for subsequent immunomodulatory effects. In addition, we found that α-melittin-NPs mediate the activation of LSECs in a concentration-dependent manner in vitro (Supplementary Fig. 7). Therefore, the threshold concentration needed to activate LSECs maybe is unachievable with melittin.

The generation and regulation of anti-tumor immunity is a highly orchestrated multistep process, which involves in the capture of tumor-associated antigens by APC, migration to draining lymph nodes, and the generation of protective T-cell response in the lymph node^39^. Unlike other APCs, LSECs do not require the time-consuming step of migration to lymphatic tissue. But the attraction and retention of specific effector populations of lymphocytes to the liver is necessary for the generation of an anti-tumor immune response, in which the chemokines and their corresponding chemokine receptors play an important role^40^. NK cells play a critical role in anti-tumor immunity and they could respond to several chemokine signals, including CCL3/CCL4/CCL5 via CCR5 and CXCL9/CXCL10 via CXCR3^40–42^. Our results showed that α-melittin-NPs increased the levels of CXCL9, CXCL10, CCL3, CCL4, CCL5, and CXCL13 as well as the recruitment of NK and T cells. In addition, the levels of cytokines IL-18 and IL-1α also increased, which is consistent with the previous reports that suggested IL-18 mediates NK-cell differentiation and maturation to suppress liver metastasis of colorectal cancer^29^ and that membrane IL-1α inhibits the development of hepatocellular carcinoma via promoting NK-cell activation^28^. Interestingly, we did not observe large increases in the level of “cytokine storm”-inducing cytokines, such as TNF-α, IL-6, IL-8, and IL-17A^43–45^, which may explain absence of liver damage induced by α-melittin-NP.

In summary, we have demonstrated that α-melittin-NPs specifically target and modulate LSECs, converting these normally tolerogenic cells into potent APCs. Once the LSECs have been activated by α-melittin-NPs, a series of cytokines and chemokines are released, recruiting a variety of innate and adaptive immune cells to the liver and generating protective T-cell immunity through coordination with NK cells to inhibit liver metastasis. Thus, LSECs could serve as an initial immunotherapy target to prevent liver metastasis. We believe that the concept of selectively breaking the tolerance properties of LSECs opens possibilities for the treatment of liver metastasis and will be successfully translated to clinical setting.

## Method

### Materials

Cholesterol oleate (CO), heparin, and protease inhibitor cocktail were purchased from Sigma-Aldrich Co. (St. Louis, MO, USA). 1,2-dimyristoyl-sn-glycero-3-phosphocholine (DMPC) was obtained from Avanti Polar Lipids Inc. (Alabaster, AL, USA). The α-peptide (DWFKAFYDKVAEKFKEAF-NH_2_) and α-melittin (DWFKAFYDKVAEKFKEAF-GSG-GIGAVLKVLTTGLPALISW-IKRKRQQ-NH_2_) were synthesized by Apeptide Co., Ltd. (Shanghai, China).

### Mice and cells

Female C57BL/6 and BALB/c mice were purchased from the Hunan SJA Laboratory Animal Co., Ltd (Changsha, Hunan, China). Actb-EGFP C57BL/6 mice were kindly provided by Dr. Zhiying He (Second Military Medical University, Shanghai, China). Mice were housed in local animal facility for at least 1 week before the experiments and used in studies when 6–8 weeks old. All of the mice were bred and maintained in a specific pathogen-free (SPF) barrier facility at Animal Center of Wuhan National Laboratory for Optoelectronics. All animal studies were conducted in compliance with protocols that had been approved by the Hubei Provincial Animal Care and Use Committee and in compliance with the experimental guidelines of the Animal Experimentation Ethics Committee of Huazhong University of Science and Technology. The B16F10 cell line was purchased from the BOSTER Company (Wuhan, China). The 4T-1 cell line was a gift from Professor Su (Huazhong University of Science and Technology, Wuhan, China). The 4T-1 and B16F10 cell lines were stably transfected with the PB transposon system (a gift from Dr. Xiaohui Wu, Fudan University, Shanghai, China), which contained the sequence encoding mCherry or mAmetrine to generate the mCherry-4T-1 and mAmetrine-B16 tumor cell lines. The CT26 cell line was obtained from ATCC. All cell lines were mycoplasma negative as determined by screening using the MycoProbe Mycoplasma Detection Kit (R and D Systems, Minneapolis, MN). These cells were cultured in RPMI-160 containing 10% FBS and 100 U/ml penicillin-streptomycin. All cells were cultured under 5% CO_2_ at 37 °C in an incubator (Thermo, USA).

### Synthesis of nanoparticles

A mixture of DMPC (3 μmol) and CO (0.2 μmol) in chloroform was dried under nitrogen to form a uniform lipid film. Then, 1 ml PBS (0.01 M, PH 7.4) was added to the dried film and the mixture was vortexed for 5 min. Subsequently, the mixture was sonicated for 1 h at 48 °C. α-melittin (0.19 μmol) or α-peptide (0.87 μmol) was dissolved in PBS and added to the lipid emulsion. The turbid emulsion immediately became transparent upon the addition of peptide. The resulting heterogenous complex peptide-associated lipid nanoparticle was stored overnight at 4 °C. After being filtered (0.22 μm) and concentrated by centrifugal filter units (30 Kd, Millipore, USA), the nanoparticles were purified using a fast protein liquid chromatography system with a HiLoad 16/60 Superdex 200 pg column (General Electric Healthcare, NY, USA) at a flow rate of 1 ml/min. The size of the eluted particles was negatively correlated with their respective retention time. Particles eluted at a retention time of ~60 min were collected as the desired nanoparticles. To prepare the nanoparticles that were core-loaded with DiR-BOA, the only difference in the protocol was in the first step, in which DiR-BOA (0.4 μmol) was mixed with the DMPC (3 μmol) and CO (0.1 μmol) in the chloroform. The peptide concentration was measured using a CBQCA protein quantitation kit (Invitrogen Corporation, CA, USA).

### Intravital imaging of LSEC targeting

The mice were anesthetized by i.p. injection of a mixture of 10 mg/kg xylazine and 100 mg/kg ketamine hydrochloride (Sigma, St. Louis, Missouri, USA). Then, the hair on the midsection was cut short with clippers and smeared with depilatory cream using cotton swabs. The depilatory cream was removed after 3 min according to the manufacturer’s instructions to avoid potential irritation to the mice. Body temperature was maintained at 37 °C using a warmer plate (Thermo Plate, TOKAI HIT, Shizuoka-ken, Japan). All the mice were surgically installed with hepatic imaging window chambers on the upper abdomen as previously described^46^. To relieve the pain of surgery, mice were intraperitoneally injected with tolfedine (16.25 mg/kg, Vétoquinol, Lavaltrie, Québec, Canada) at the end of the procedure. After 24 h, the mice were used in the imaging experiments. First, the mice were anesthetized with isoflurane inhalation [1.5–2% (v/v) isoflurane in O_2_] and placed within a custom-designed imaging box. The isoflurane was introduced through a rubber tube and ventilated by an outlet on the other side of the box. The imaging window was placed on a hole in the bottom of the box. Then the body of the mouse was fixed to the box using medical tape, and the tail was pulled out of box for convenient intravenous injection of the nanoparticles. Real-time and long-term targeting assays were conducted using an Olympus IX83 confocal microscope outfitted with an UltraVIEW VoX 3D live cell imaging system (PerkinElmer). Fluorescence was observed using a ×20/0.75 NA objective or a ×60 apochromat 1.42 NA oil objective. All fluorescence images were acquired using Volocity 6.3 (PerkinElmer) and analyzed with Image J software (National Institutes of Health). Movie was further edited with Adobe Premier Pro CC version 2017 1.1.

### Liver metastasis model

To induce the experimental liver metastasis models, mice were anesthetized by i.p. injection with a mixture of 10 mg/kg xylazine and 100 mg/kg ketamine hydrochloride (Sigma, St. Louis, Missouri, USA), and 2 × 10^5^ tumor cells were inoculated into the exposed spleen of the mice (B16F10 cells in C57BL/6 mice, 4T-1, and CT26 cells in BALB/c mice). Seven minutes later, the half of the spleen that received the cells was resected to decrease primary tumor growth in the spleen, and the small incision was closed. Body temperature was maintained at 37 °C using a warmer plate (Thermo Plate, TOKAI HIT, Shizuoka-ken, Japan) for recovery until the mouse was mobile demonstrated regular breathing patterns. Mice were killed on day 15 for the B16F10 model and on day 21 for the 4T-1 or CT26 models. To induce the spontaneous liver metastatic model, 3 × 10^5^ mCherry-4T-1 cells in 100 μl of PBS were inoculated into the mammary fat pads of syngeneic BALB/c mice, and the primary tumors were resected when they reached 450 mm^3^. The mice were given treatments of PBS, α-peptide-NPs, or α-melittin-NPs on days 0, 7, and 14, respectively, after the operation. The mice were killed, and the organs were collected on day 40 after primary tumor resection. To quantify the liver metastatic burden, H&E slides were scanned with a Nikon Ni-E (Nikon, Minato, Tokyo, Japan) and images were acquired with NIS-Elements software and further analyzed with ImageJ. The metastatic burden was calculated by dividing the area occupied by metastatic foci (mm^2^) by the total surface liver area (mm^2^).

### Non-parenchymal liver cell preparation

After the mice were anesthetized, the liver and portal vein were exposed via a ventral midline incision, and a 22 G intravenous catheter was inserted into the portal vein. The liver was perfused at 7 ml/min via the portal vein for 10 min with Gey’s balanced salt solution (GBSS) without Ca^2+^ at 37 °C until the liver was completely discolored and then for 10 min with GBSS containing 0.1 mg/ml collagenase IV (Worthington). After the two-step collagenase perfusion, the liver was excised, finely smashed with forceps in perfusion buffer, and placed in a dish containing 0.1 mg/ml collagenase IV and 0.02 mg/ml DNase I (Sigma-Aldrich) at 37 °C for 10 min. The liver cell suspension was collected, and parenchymal cells were separated from non-parenchymal cells (NPCs) by centrifugation for 2 min at 50 g. The supernatant containing the NPCs was collected and centrifuged for 10 min at 350×*g*. The pellet was then resuspended in PBS and layered (3.3 ml) on the top of a 2-step Percoll gradient (5 ml of 50% Percoll in the bottom and 6.6 ml of 25% Percoll in the top). The gradients were centrifuged for 20 min at room temperature at 750 g, and the intermediate layer containing NPCs was collected. To analyze the subhepatic distribution of nanoparticles, the NPC fraction was suspended in 2–3 ml of ice-cold PBS for flow cytometry (FCM). For the transcriptome RNA-seq analysis of LSECs, LSECs were separated from NPCs using anti-CD146 immunomagnetic beads (Miltenyi Biotec, Bergisch-Gladbach, Germany) according to the manufacturer’s instruction. For the in vitro experiments, the isolated LSECs were seeded on rat tail collagen-coated plates in microvascular endothelial cell media-2 (EGM-2 MV) medium (Lonza, Basel, Switzerland).

### RNA extraction and real-time RT-PCR

Total RNA was extracted from mouse liver tissues or cultured LSECs using the TRIzol reagent (Invitrogen, Carlsbad, CA). Equal amounts of RNA were reverse transcribed into cDNA using the PrimeScript RT Reagent Kit with gDNA Eraser (Takara, Dalian, China) and the real-time RT-PCR assay was performed using the StepOnePlus™ Real-Time PCR System (Applied Biosystems). The mRNA level of the targeted genes was normalized to that of actin. The data were analyzed by the relative quantification (ΔΔCt) method. Primer sequences are listed in Supplementary Table 2.

### Isolation of intrahepatic leukocytes

The liver was minced into 1 mm pieces and digested using collagenase IV (Worthington) for 30 min at 37 °C. The digested liver extracts were filtered through 70-μm cell strainers and centrifuged at 500×*g* for 5 min. The resulting cell pellet was resuspended in 10 ml of 35% Percoll containing 100 U/ml heparin and centrifuged at 700×*g* for 15 min at room temperature. The cell pellet containing the leukocytes were collected and resuspended in 3 ml of red blood cell lysis solution (155 mM NH_4_Cl, 10 mM KHCO_3_, 1 mM EDTA, 170 mM Tris, pH 7.3). After incubation for 3 min on ice, the cells were washed twice in RPMI 1640 containing 5% fetal bovine serum.

### Flow cytometry

The antibodies to CD45 (Clone: 104/30-F11, Catalog: 104522/103108), CD3 (Clone: 17A2, Catalog: 100204), NK1.1 (Clone: PK136, Catalog: 108710), CD69 (Clone: H1.2F3 Catalog: 104522), CD11b (Clone: M1/70, Catalog: 101212), CD27 (Clone: LG.3A10, Catalog: 124209), Ki-67 (Clone: 16A8, Catalog: 652411), Ly6G (Clone: 1A8, Catalog: 127624), Ly6C (Clone: HK1.4, Catalog: 128006), F4/80 (Clone: BM8, Catalog: 123132), CD8 (Clone: 53–6.7, Catalog: 100722), CD19 (Clone: 6D5, Catalog: 115507), CD4 (Clone: RM4–4, Catalog: 116012), CD146 (Clone: ME-9F1, Catalog: 134714), CD11c (Clone: N418, Catalog: 117334), MHC-II (Clone: M5/114.15.2, Catalog: 107626), CD86 (Clone: GL-1, Catalog: 105011), CD80 (Clone: 16-10A1, Catalog: 104722), Tim3 (Clone: RMT3-23, Catalog: 1197237), ICOS (Clone: HK5.3, Catalog: 117405), CD62L (Clone: MEL-14, Catalog: 104418), and CD44 (Clone: IM7, Catalog: 103032) were purchased from BioLegend. The Antibody to granzyme B (Clone: NGZB, Catalog: 12-8898-80) and fixable viability dye eFluor506 (Catalog: 65-0866-18) were purchased from eBioscience. Cells isolated from liver were processed for surface labeling with appropriate antibodies according to the manufacturer’s instructions. Viability was assessed by the fixable viability dye eFluor506. The cells were further permeabilized using the Transcription Factor Buffer Set (BioLegend) and stained for Ki-67 and granzyme B. All the cells were analyzed using a CytoFLEX flow cytometer (Beckman Coulter, USA). The data were analyzed using FlowJo software (FlowJo, Ashland, OR, USA).

### RNA-seq and bioinformatic data analysis

LSECs were isolated as described above, and the total RNA was extracted using TRIzol (Invitrogen, Carlsbad, CA). Total RNA (2 μg) was used for the stranded mRNA sequencing library preparation using the VAHTS mRNA-seq v2 Library Prep Kit for Illumina (Vazyme biotech Co., Nanjing, China), following the manufacturer’s instructions. The PCR products that were 200–500 bps in length were purified, quantified, and finally sequenced on a HiSeq X10 sequencer (Illumina). For the RNA-seq data analysis, raw sequencing data (309 million raw paired-end reads) were first filtered by Trimmomatic (version: 0.36); low-quality reads were discarded, and adaptor sequences were trimmed. After quality filtering, each sample had ~30.2–35.8 million clean reads. Clean reads from each sample were mapped to the GRCm38 mouse reference genome using the Star program (2.3.0). A corrected *P*-value cutoff of 0.05 and Fold change cutoff of 1 were used to judge the statistical significance of differences in gene expression. GO analysis of differentially expressed genes was conducted using the GO-seq R package, with a corrected *P*-value cutoff of 0.05 to determine statistically significant enrichment. KEGG enrichment analysis of DEGs was implemented by KOBAS software (version: 2.1.1).

### Cytokine and chemokine quantitation

The livers were collected, and their masses were measured at 24 h after treatment. Then, tissue samples were lysed in lysis buffer (5 μl/mg liver) containing 50 mM Tris-HCl (pH 7.5), 10% glycerol, 150 mM NaCl, and 1% NP-40 and freshly supplemented with protease inhibitor cocktail (Sigma-Aldrich). Lysates were aliquoted and stored at −80 °C until analysis. Samples were assayed using the LEGENDplex^TM^ mouse inflammation and chemokine panel array (BioLegend) according to the manufacturer’s instructions. The data were analyzed with Legendplex software (BioLegend). For the in vitro experiments, the LSECs cell media were collected at 24 h after treatments for the detection of IL-1α, IL-18, and CXCL10 using ELISA kits according to the manufacturer’s instructions (Neobioscience, Shenzhen, China).

### Biochemical analyses

Blood samples were collected before the mice were killed. The biochemical analyses were performed using a biochemical analyzer (SPOTCHEM EZ SP-4430, Arkray Inc., Kyoto, Japan).

### Immunofluorescence staining

For the immunofluorescence analysis, liver tissues were fixed in 4% paraformaldehyde for 12 h at 4 °C and then dehydrated in 30% sucrose solution. The tissues were then frozen in OCT (Sakura, Torrance, CA, USA) compound and sectioned into 10 μm slices using a freezing microtome (Leica, Germany). OCT was removed by washing three times in PBS, and the sections were immunostained with Alexa Fluor 647 anti-mouse NK1.1 (BioLegend, Clone: PK136, Catalog: 108720) or CD8 (BioLegend, Clone: 53–6.7, Catalog: 100724) at 1:200 dilution. All the sections were imaged with LSM 710 laser confocal scanning microscopy (Zeiss, Germany). The data were analyzed using ImageJ software.

### Statistical analysis

A one-way ANOVA followed by a post hoc test was used for multiple group comparisons, and Student’s *t*-test (two-tailed) was used for comparisons of two groups. A log-rank (Mantel-Cox) test was used for survival data. All statistical analyses were performed with GraphPad Prism 6 (GraphPad Software, CA, USA). Values were presented as mean ± SEM. Differences were considered statistically significant at *P < 0.05, **P < 0.01, ***P < 0.001, and ****P < 0.0001.

### Reporting summary

Further information on experimental design is available in the Nature Research Reporting Summary linked to this article.

## Supplementary information

Supplementary Information

Description of Additional Supplementary Files

Supplementary Movie 1

Reporting Summary

## Data Availability

Sequencing data have been deposited in Short Read Archive under project number PRJNA503822. The authors declare that all other data supporting the findings of this study are available within the article and its Supplementary Information files or from the corresponding authors on reasonable request.
