# Supplementary material for: Immune modulation of liver sinusoidal endothelial cells by melittin nanoparticles suppresses liver metastasis
Source: Nat Commun. 2019 Feb 4;10:574. doi: 10.1038/s41467-019-08538-x (PMC6361944; doi:10.1038/s41467-019-08538-x)
Supplement: Supplementary file 1 — Supplementary Information [file 41467_2019_8538_MOESM1_ESM.pdf]

Supplementary Information for

Immune Modulation of Liver Sinusoidal Endothelial Cells by Melittin Nanoparticles Suppresses Liver Metastasis

Yu et al.

This PDF file includes:

Supplementary Figure 1 to 12 and Legends

Supplementary Table 1 and 2

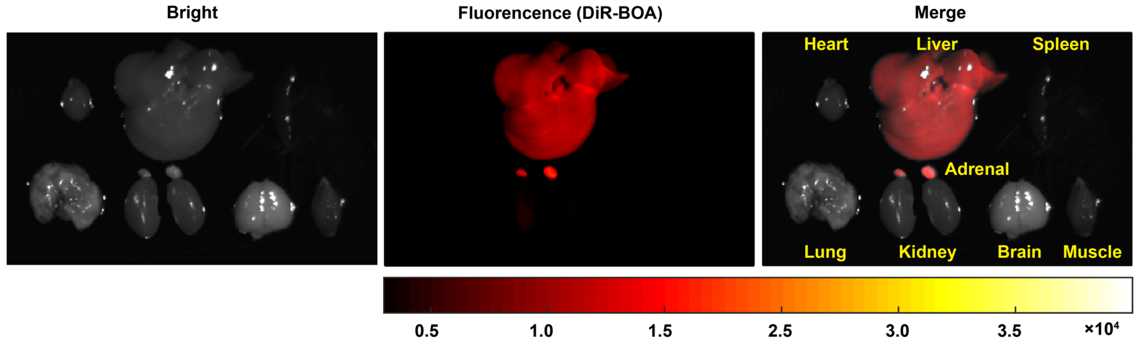

Supplementary Figure 1: Representative fluorescence imaging of the biodistribution of the α-melittin-NPs. The (DiR-BOA)α-melittin-NP (20 μM) was intravenously injected into C57BL/6 mice, and the different organs were dissected at 6 h post-injection. Fluorescence images of DiR-BOA (red) were acquired with an NIR filter set (excitation: 716/40 nm; emission: 800/40 nm).

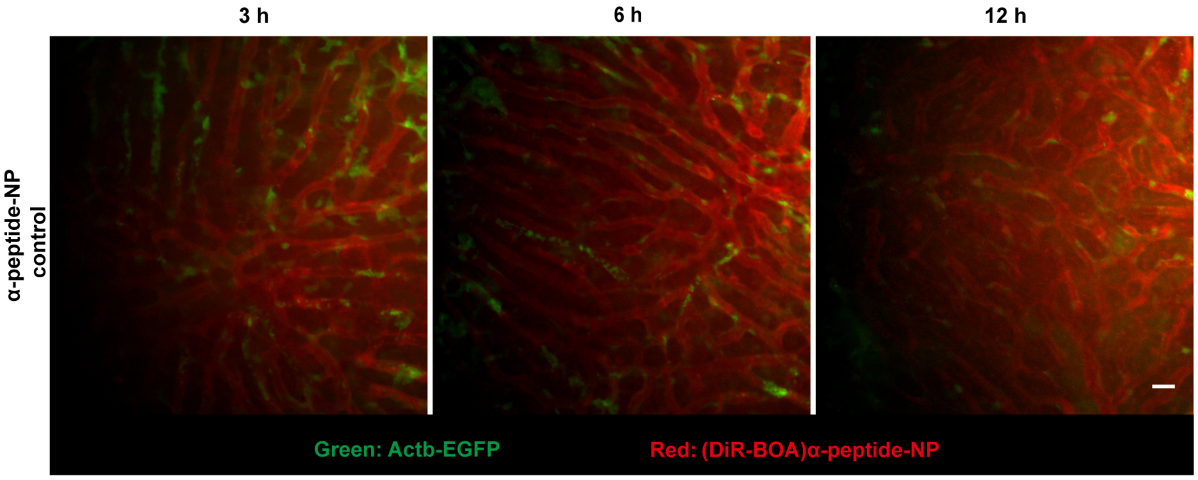

Supplementary Figure 2: Long-term intravital imaging of the distribution of the α-peptide-NP control. α-peptide-NPs were labeled with DiR-BOA (red), a lipid-anchored near-infrared fluorophore. The Actb-EGFP mice were used to visualize the structure of the liver. Images are representative of 3 trials. Scale bar, 20 μm.

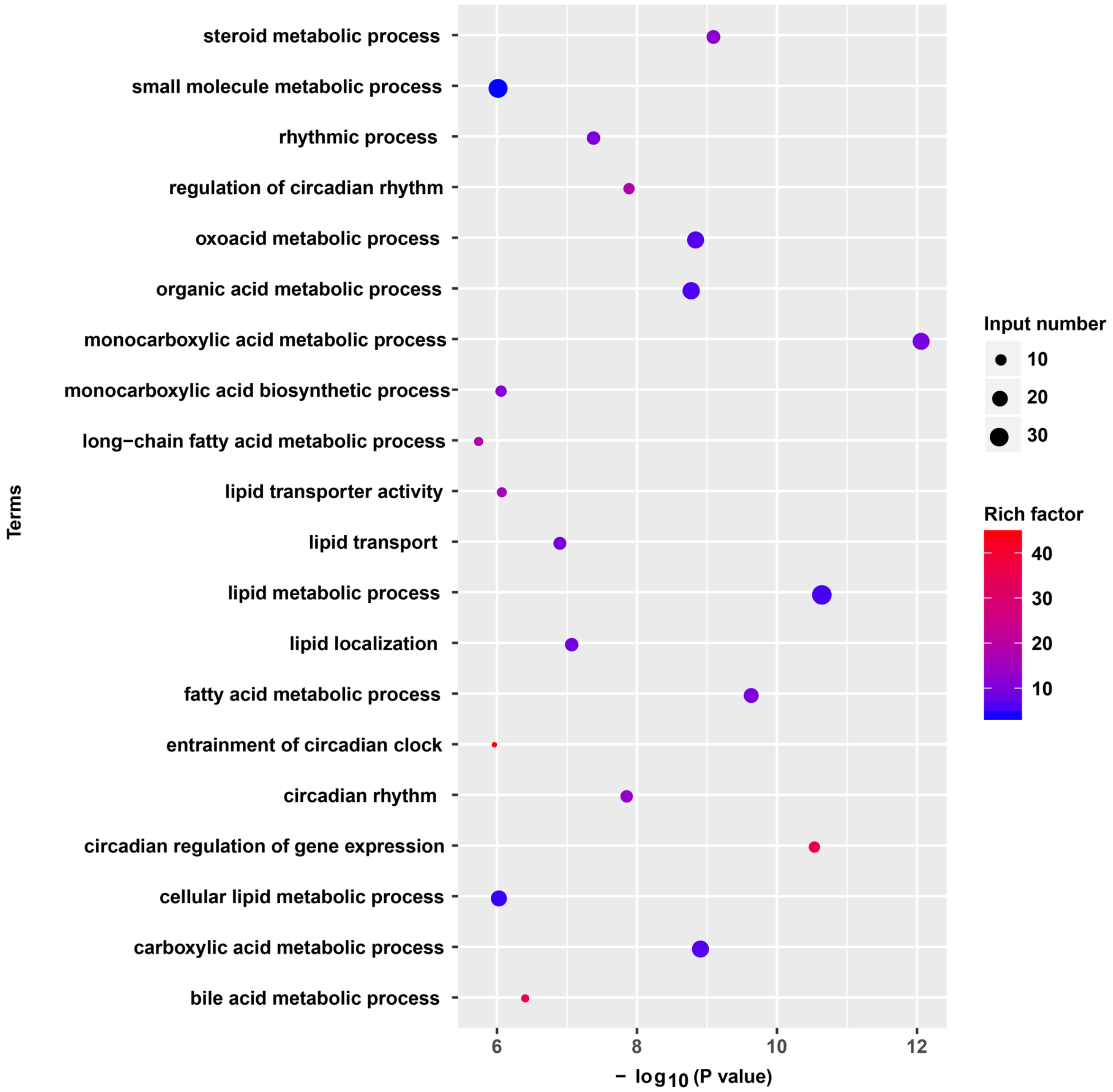

Supplementary Figure 3: Scatter plot of the GO enrichment results for the down-regulated genes of LSECs. In relation to figure 2, GO enrichments for down-regulated genes are shown here. The dot size indicates the number of DEGs contained in the GO terms, and the dot depth indicates the extent of the rich factor enrichment.

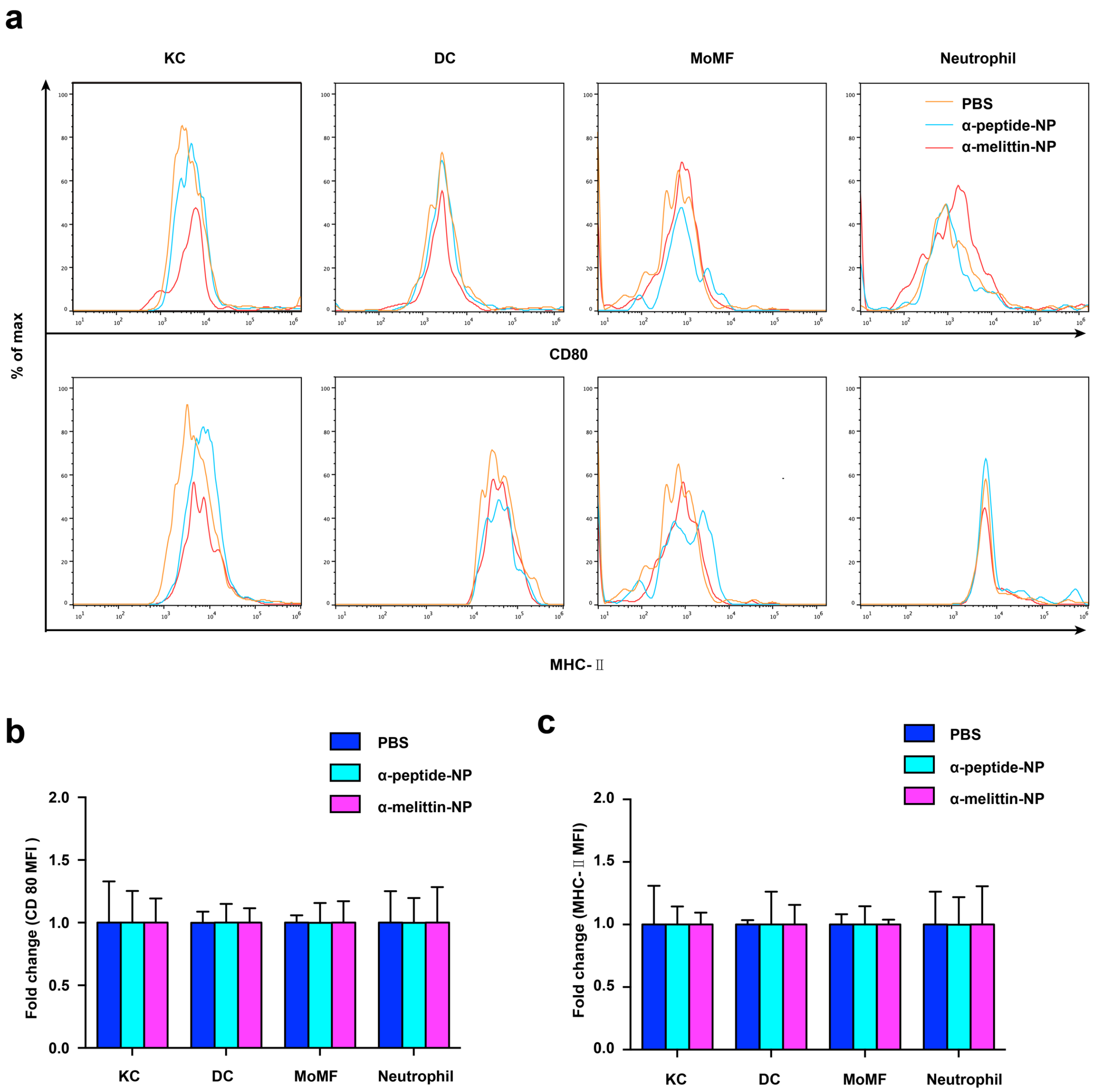

Supplementary Figure 4: The effect of α-melittin-NPs on other phagocytic cells. (**a**) Representative histograms of CD80 and MHC-II expression on KCs, DCs, MoMFs, and neutrophils. (**b, c**) Quantitative data of the MFIs of CD80 (**b**) and MHC-II (**c**) in different cell populations. Error bars indicate SEM. Statistical analysis was performed with one-way ANOVA followed by Bonferroni's post hoc test.

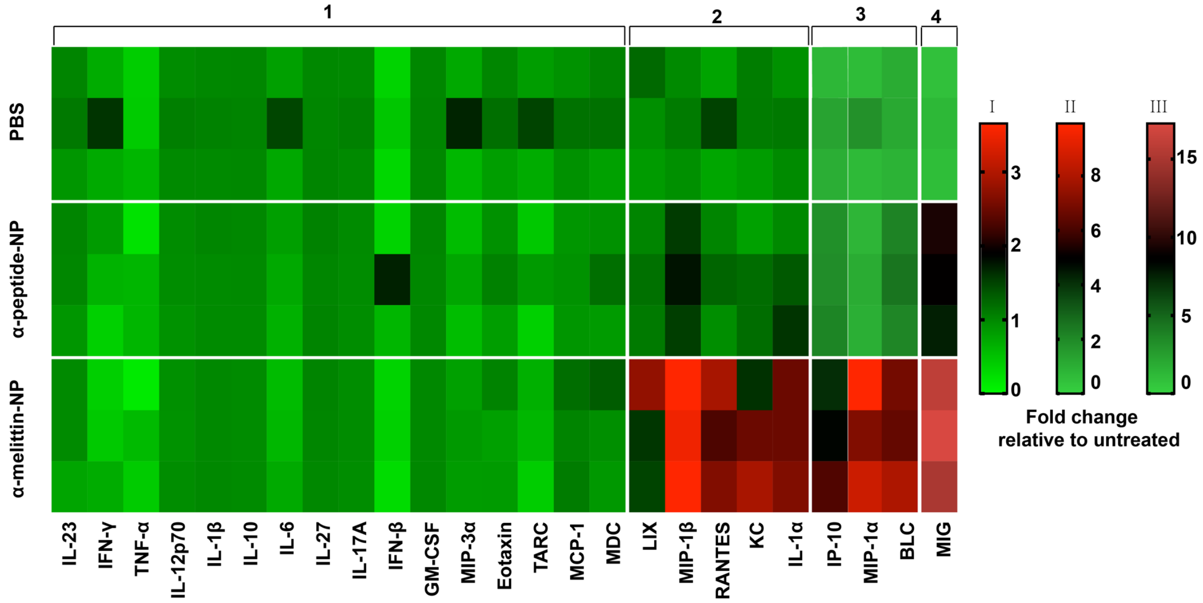

Supplementary Figure 5: α-melittin-NPs induce the dramatic changes of the cytokines/chemokines protein abundance in the liver. Liver tissue samples were collected and lysed in lysis buffer (5 μl/mg) at 24 h after treatment. The levels of the pro-inflammatory cytokines and chemokines were measured by LEGENDplexTM mouse inflammation and chemokine panel (13-plex) arrays (n = 3 mice per group). The levels of cytokines and chemokines were divided into four clusters according to the fold change relative to the untreated group. Clusters 1 and 2 share bar Ⅰ, while clusters 3 and 4 are represented by bars Ⅱ and Ⅲ, respectively.

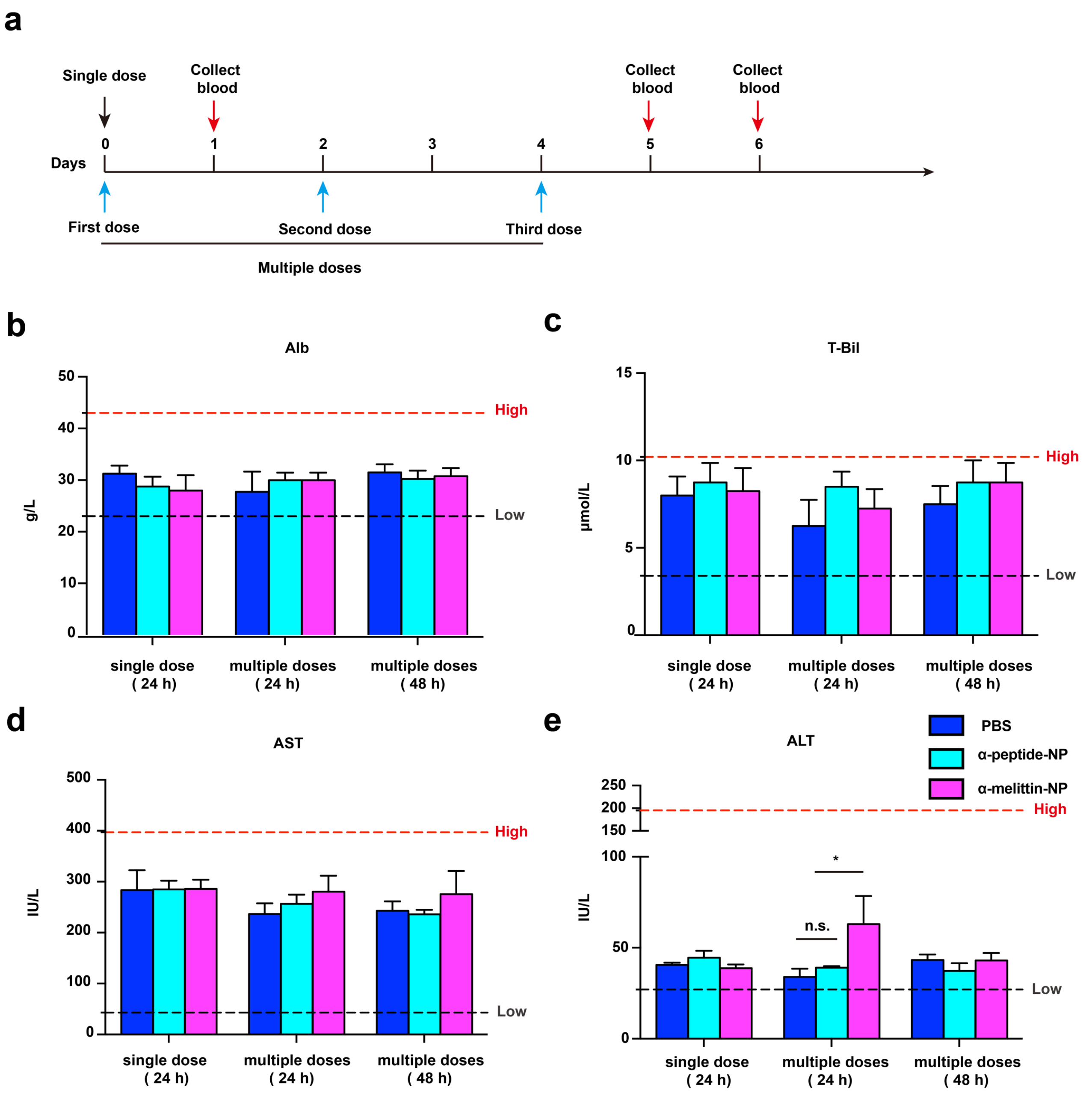

Supplementary Figure 6: Evaluation of liver function after α-melittin-NPs administration. After different administrations, the blood samples from the mice (n = 4 per group) were collected and liver function markers, including albumin (Alb), total bilirubin (T-Bil), aspartate aminotransferase (AST), and alanine aminotransferase (ALT), were measured and are shown in Supplementary Figure 6 b–e, respectively. Reference ranges of the markers for healthy female C57BL/6 mice were obtained from Charles River Laboratories: (http://www.criver.com/). The dotted black line indicates the minimum value of the reference range, and the dotted red line indicates the maximum value. Error bars indicate SEM. n.s., not significant; *$P < 0.05$; by one-way ANOVA followed by Bonferroni's post hoc test.

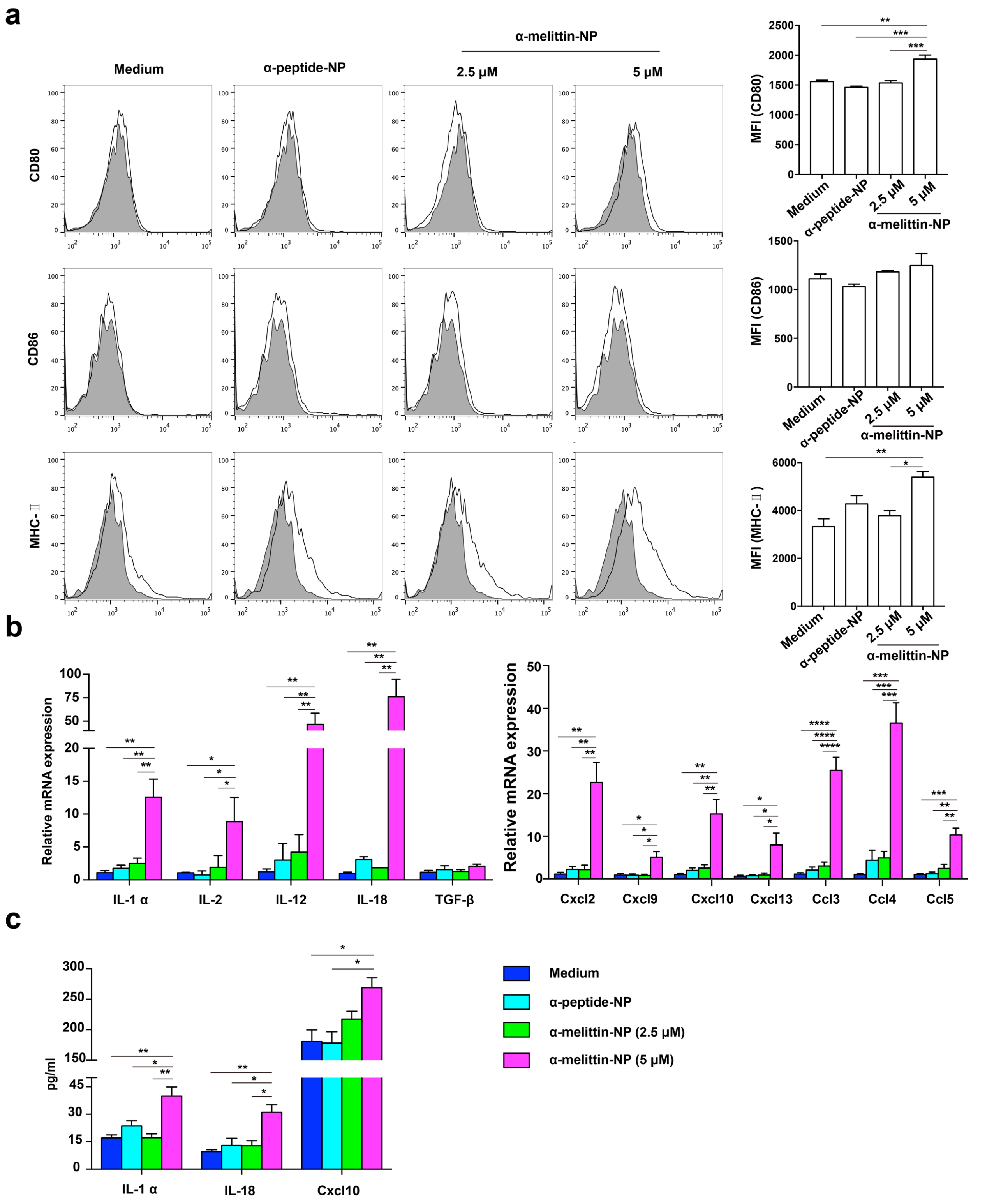

Supplementary Figure 7: In vitro demonstration of the concentration-dependent activation of LSECs by α-melittin-NPs. (**a**) The expression of surface markers on LSECs isolated from C57BL/6 mice at 24 h after different treatments. (**b**) The mRNA expression levels of cytokines/chemokines in the LSECs at 3 h after stimulations. (**c**) The protein levels of selected cytokines/chemokines at 24 h after different treatments. Throughout, n = 3 per group. Error bars indicate SEM. n.s., not significant; ****P < 0.0001; ***P < 0.001; **P < 0.01; *P < 0.05; by one-way ANOVA followed by Bonferroni's post hoc test.

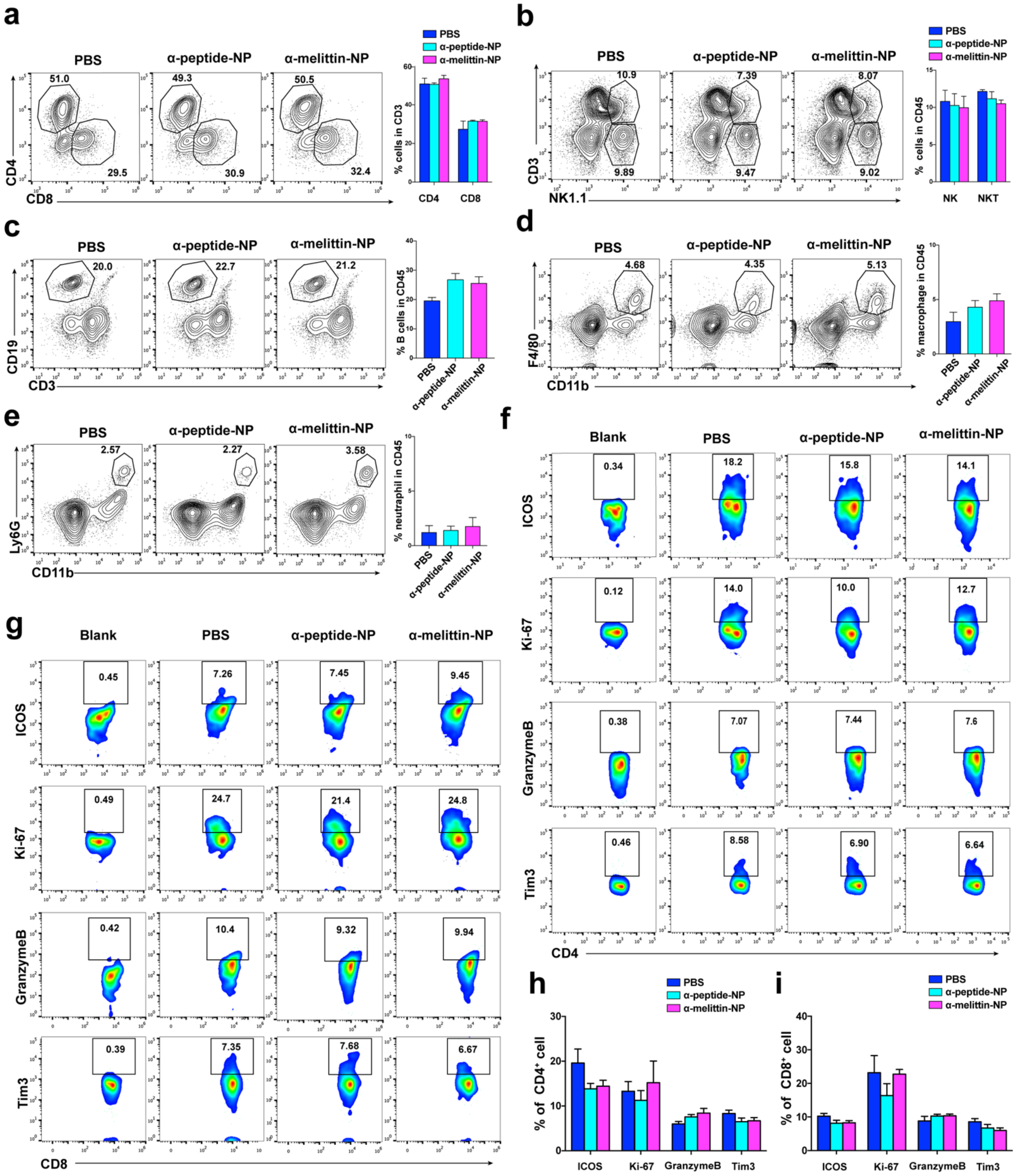

Supplementary Figure 8: Characterization of immune cell subsets in the no tumor-bearing mice after α-melittin-NP treatment. Related to figure 2, 24 h after different treatments, hepatic lymphocytes were collected at by *in vivo* perfusion and *in vitro* digestion to assess the immune cell infiltration in the liver. (**a–e**) The percentages of $CD4^+$ and $CD8^+$ T cells (**a**), NK ($NK1.1^+CD3^-$) and NKT cells ($NK1.1^+CD3^+$) (**b**), B cells (**c**), macrophages ($CD11b^+F4/80^+$)(**d**), and neutrophils ($CD11b^+Ly6G^+Ly6C^-$) (**e**). (**f–i**) Representative FCM plots and cumulative results for $CD4^+$ T cells (**f, h**) and $CD8^+$ T cells (**g, i**) according to the expression of ICOS, Ki-67, granzyme B, and Tim3. Error bars indicate SEM. Statistical analysis was performed with one-way ANOVA followed by Bonferroni's post hoc test.

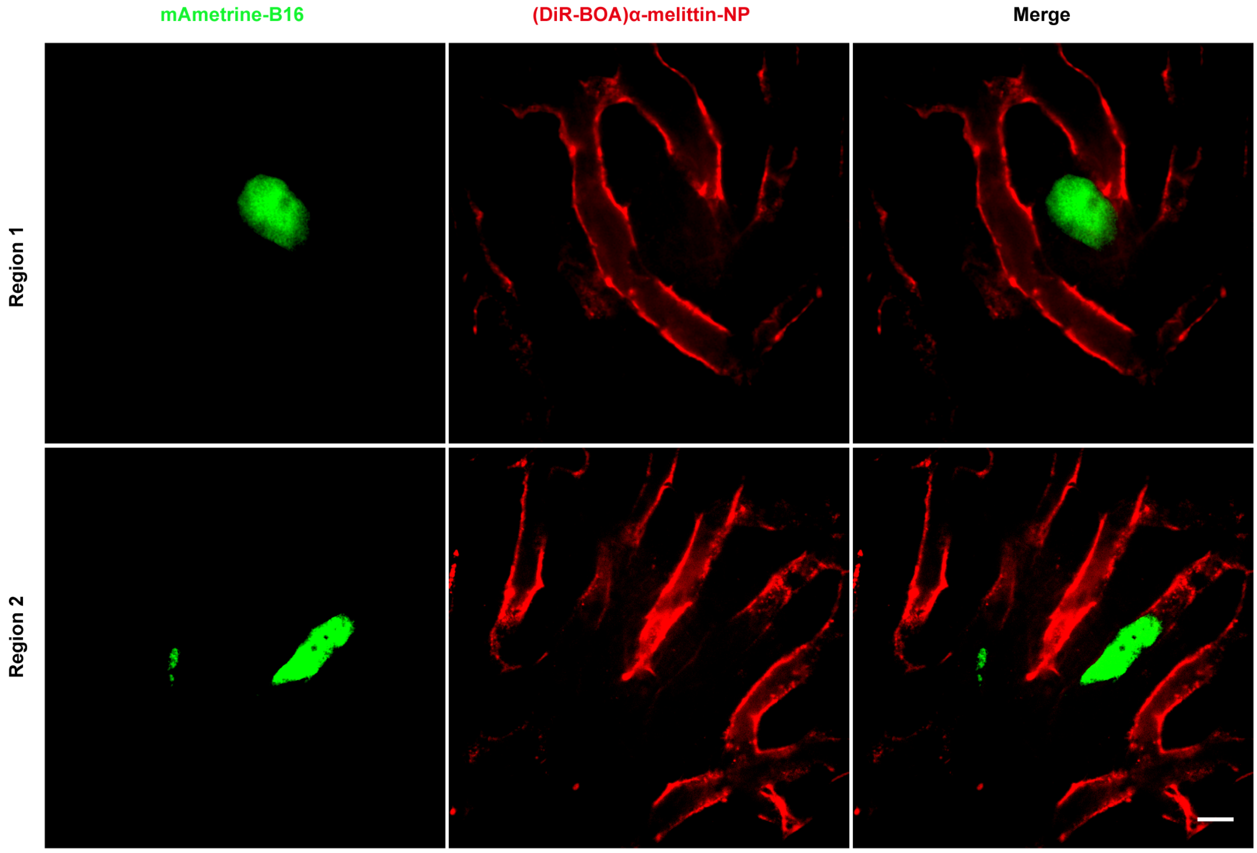

Supplementary Figure 9: Metastatic tumor cells rapidly arrived at the liver after inoculation. The B16 tumor cells were identified with mAmetrine (green), which is a long stokes shift variant of Aequorea GFP and retains the property of violet excitation (excitation wavelength, λex = 406 nm, emission wavelength, λem = 526 nm), thus avoiding interference with the autofluorescence of the liver at 488 nm. α-melittin-NPs were identified using DiR-BOA (red). Mice were surgically installed with hepatic imaging window chambers and the liver was observed by spinning disk confocal microscope 3 hours after tumor inoculation. Images are two representative regions of 3 trials. Scale bar, 20 μm.

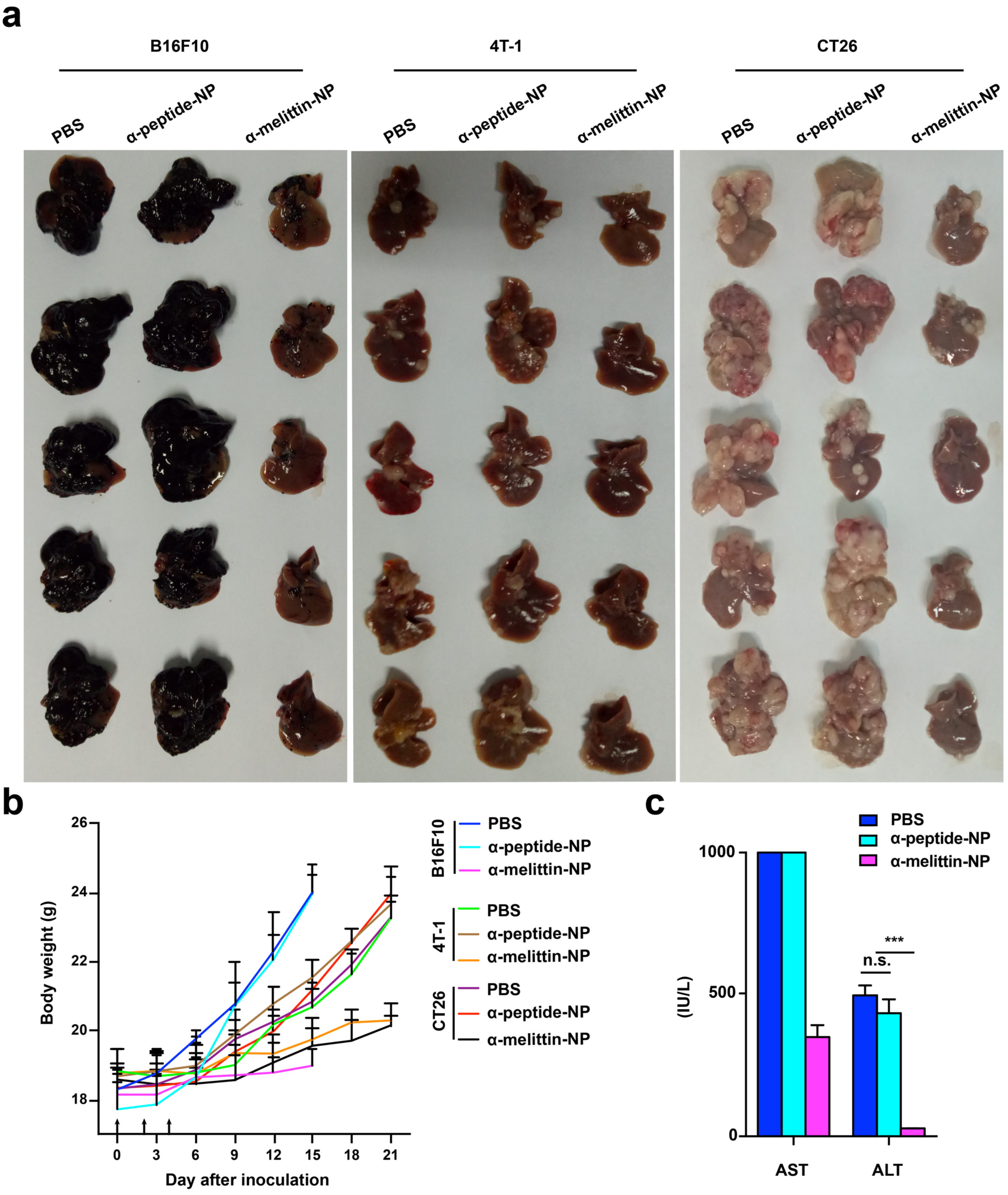

Supplementary Figure 10: The photo image of liver after α-melittin-NP therapy. Related to figure 3, groups of mice (n = 5 mice/group) were injected with 2 × 10^5 B16F10, 4T-1 or CT26 in the hemispleen and then treated with PBS, α-peptide-NPs or α-melittin-NPs according to the timeline in **Fig. 3a**. At the end of the treatment period, blood samples were collected to analyze the biochemical indicators of hepatotoxicity. (**a**) Images of livers from each group. (**b**) Body weight measurements over time from three different tumor models; the black arrows indicate the treatment time points. (**c**) Analysis of ALT and AST levels in the sera of tumor-bearing mice at the end of treatment in the B16 tumor model. The level of AST in the PBS and α-peptide-NP groups exceeded the instrument detection limits. Error bars indicate SEM. n.s., not significant; ***$P < 0.001$; by one-way ANOVA followed by Bonferroni's post hoc test.

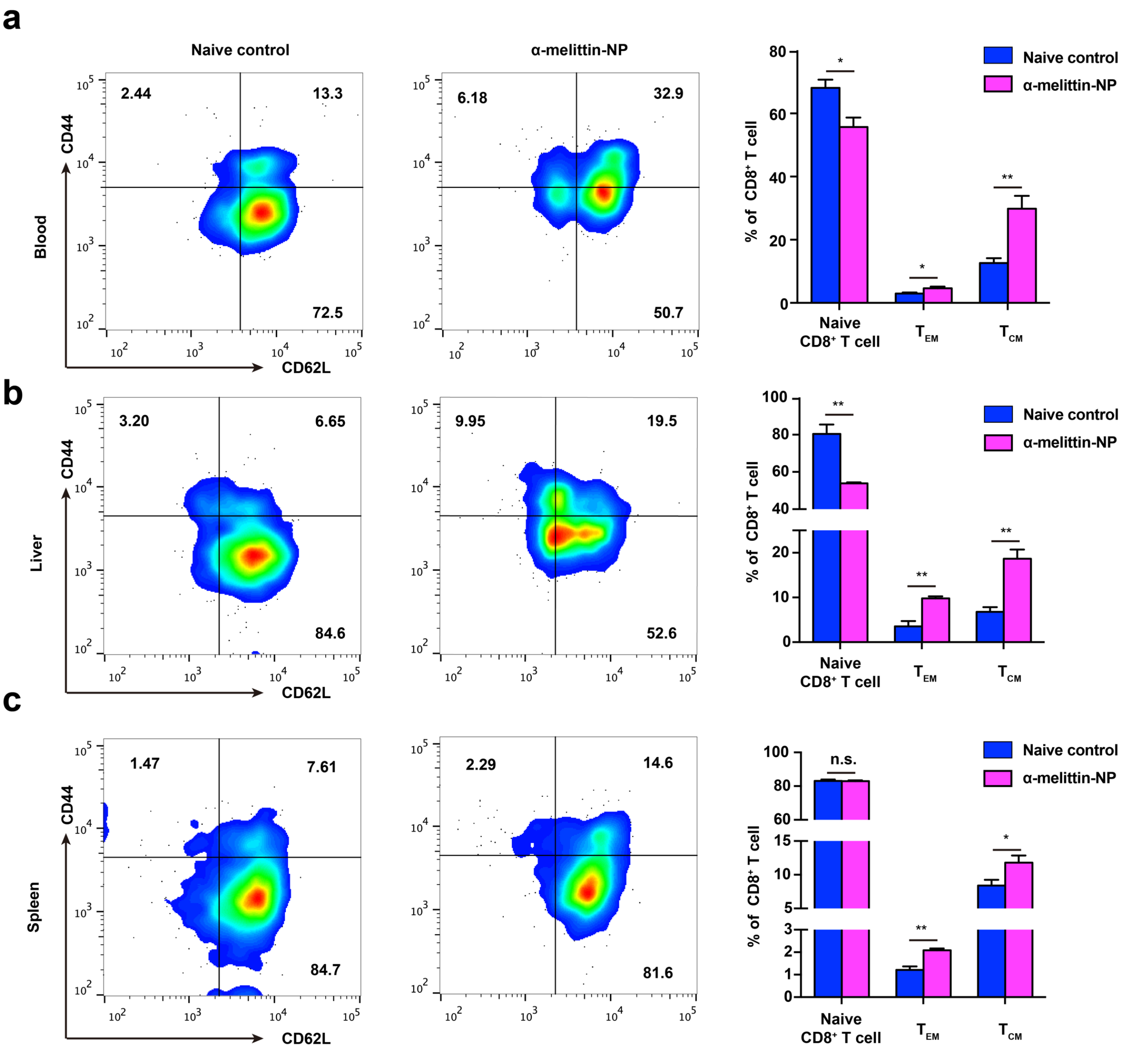

Supplementary Figure 11: Immune memory effects of α-melittin-NPs. (**a**–**c**) The percentages of effector memory cells (T_{EM}) cells and central memory (T_{CM}) cells in the blood (**a**), liver (**b**), and spleen (**c**) at day 28 after treatment. Representative FCM plots and cumulative results are shown in left and right panels， respectively. Throughout, n = 4 per group. Error bars indicate SEM. n.s., not significant; **P < 0.01; *P < 0.05; by Student's t test.

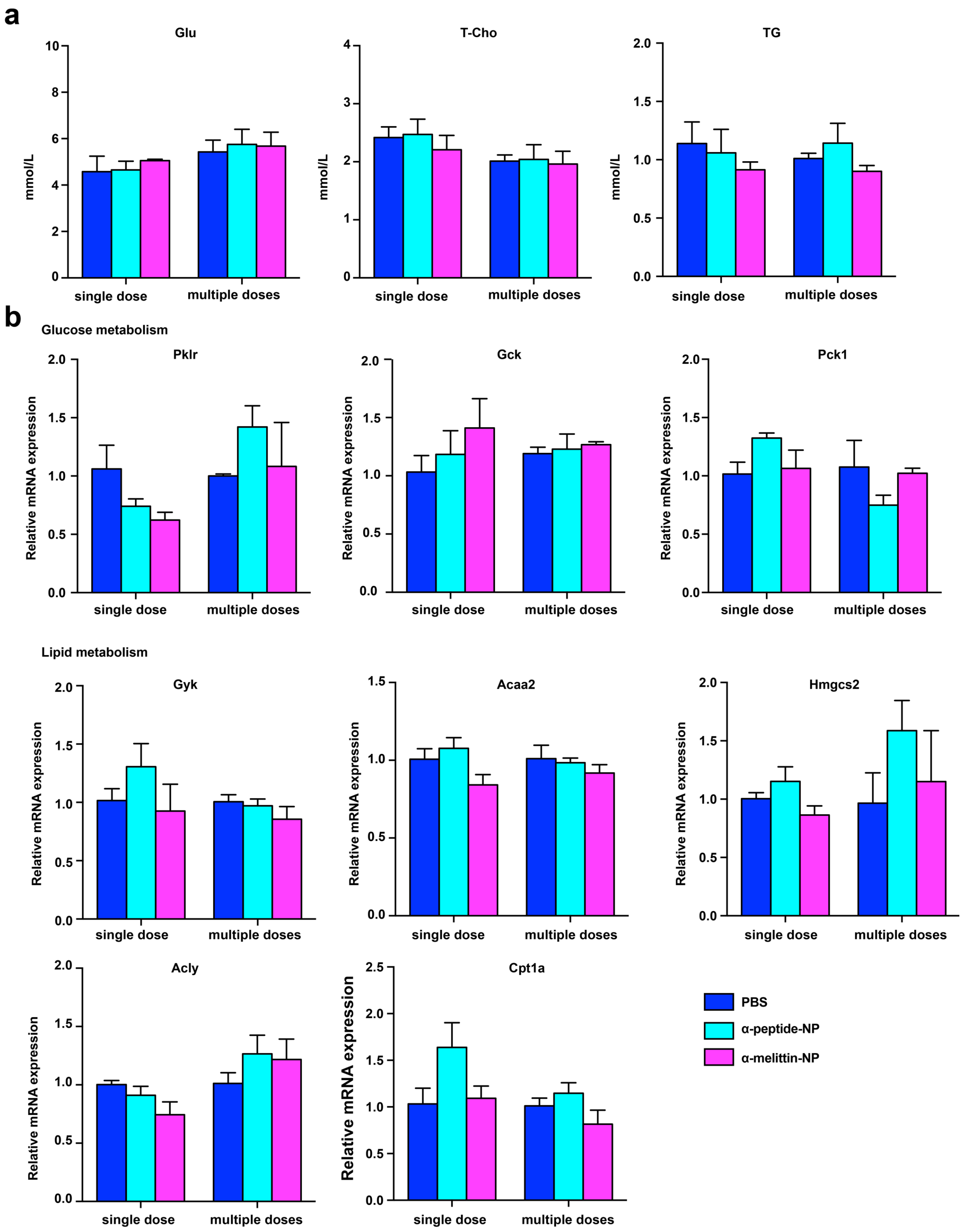

Supplementary Figure 12: Evaluation of the effect of α-melittin-NPs on liver metabolism. (**a**) Abundance of glucose (Glu), total cholesterol (T-Cho), and triglyceride (TG) after different treatments (n = 4 mice per group). (**b**) Gene expression levels of rate-limiting enzymes in glycolysis [pyruvate kinase (Pklr) and glycolysis (Gck)], gluconeogenesis [phosphoenolpyruvate carboxykinase (Pck1)], triglyceride synthesis [glycerol kinase (Gyk)], cholesterol synthesis [acetyl-coenzyme A acyltransferase 2 (Acaa2) and HMG CoA synthase 2 (Hmgcs2)], fatty acid synthesis [ATP citrate lyase (Acly)], and fatty acid β oxidation [carnitine palmitoyltransferase 1a (Cpt1a)]. Error bars indicate SEM. Statistical analysis was performed with one-way ANOVA followed by Bonferroni's post hoc test.

Supplementary Table 1: Rates of primary tumor recurrence and organ metastasis.

	PBS	α-peptide-NP	α-melittin-NP
Primary tumor (Recurrence)	100% (5/5)	100% (5/5)	0% (0/5)
Liver	80% (4/5)	80% (4/5)	0% (0/5)
Lung	100% (5/5)	100% (5/5)	0% (0/5)
Lymph node	100% (5/5)	100% (5/5)	20% (1/5)
Kidney	20% (1/5)	0% (0/5)	0% (0/5)
Heart	20% (1/5)	0% (0/5)	0% (0/5)
Spleen	0% (0/5)	0% (0/5)	0% (0/5)
Brain	0% (0/5)	0% (0/5)	0% (0/5)

Supplementary Table 2: List of primers used in this study.

mRNA	Forward (5´–3´)	Reverse (5´–3´)
Actin	ACTCCTAAGAGGAGGATGGTCG	CAGACCTGGGCCATTCAGAAAT
IL-1α	GCTTGAGTCGGCAAAGAAATCA	CAGAGAGAGATGGTCAATGGCA
IL-2	ACTGTTGTAAAACTAAAGGGCTCTG	GCAGGAGGTACATAGTTATTGAGGG
IL-12	GCGAGAGAGGCAGAGAACTCA	GTAAGCAACCGACTCTCCCCA
IL-18	AGTTTACAAGCATCCAGGCACAG	ATGCTGGAGGTTGCAGAAGAT
TGF-β	GCTGAACCAAGGAGACGGAA	GTTTGGGGCTGATCCCGTTG
Cxcl2	GCGGTCAAAAAGTTTGCCTTGA	AGGCTCCTCCTTTCCAGGTC
Cxcl9	TCGGACTTCACTCCAACACAG	AGGGTTCCTCGAACTCCACA
Cxcl10	CCGCTGCAACTGCATCCATA	ATCGTGGCAATGATCTCAACAC
Cxcl13	ACTGAAGTTGTGATCTGGACC	AGATGATAGTGGCTTCAGGCAG
Ccl3	GCCAGGTGTCATTTTCCTGACT	CAGGAATGTTCCGGGGCTCA
Ccl4	GTGCTAACCCCAGTGAGCC	GAGCAAGGACGCTTCTCAGT
Ccl5	TGCAGTCGTGTTTGTCACTC	CAAGCAATGACAGGGAAGCTAT
Pklr	TGCACGACTCAACTTCTCCC	GGACTCCAGTGCGTATCTCGC
Gck	CTTGGCTGCCTGTCTTTTGC	CTGCTCTACCAGAGTCAACGAC
Pck1	TTGAACTGACAGACTCGCCC	TTGATGAACTCCCCATCTCCC
Gyk	TCTCATAGCCTGAAAGCTGGG	AGCACCCTGTTCCATACGTG
Acaa2	GGCAAACAGACCATGCAAGT	GTCTTTCTTGAACACGGACG
Hmgcs2	CTATGCAGCCTACCGCAAGA	TGAACATCAACCGAGCCAGG
Acly	GACCATCATTGGGCCAGCTA	AGACATGCCTCCTGAACGTG
Cpt1a	CACTGCAGCTCGCACATTAC	CCAGCACAAAGTTGCAGGAC
