# Supplementary material for: Immune modulation of liver sinusoidal endothelial cells by melittin nanoparticles suppresses liver metastasis
Source: Nat Commun. 2019 Feb 4;10:574. doi: 10.1038/s41467-019-08538-x (PMC6361944; doi:10.1038/s41467-019-08538-x)
Supplement: Supplementary file 2 — Description of Additional Supplementary Files [file 41467_2019_8538_MOESM2_ESM.docx]

**Description of Additional Supplementary Files**

**File Name:** Supplementary Movie 1

**Description:** In vivo real-time imaging of α-melittin-NPs to target the LSECs. α-melittin-NPs or α-peptide-NPs core-loaded with DiR-BOA (red), a lipid-anchored near-infrared fluorophore, were used to monitor their distribution in liver. In addition, we used Actb-EGFP mice to display the structure of the hepatic lobule and the cells in liver sinusoid via intravital imaging. The data showed that the boundaries of hepatic sinusoid were clearly delineated by α-melittin-NPs, whereas the control nanoparticles (α-peptide-NPs) flowed through the hepatic sinusoid and rarely labeled the LSECs. Time is indicated as min:sec. Scale bar, 20 μm.
