# Supplementary material for: Immune modulation of liver sinusoidal endothelial cells by melittin nanoparticles suppresses liver metastasis
Source: Nat Commun. 2019 Feb 4;10:574. doi: 10.1038/s41467-019-08538-x (PMC6361944; doi:10.1038/s41467-019-08538-x)
Supplement: Supplementary file 4 — Reporting Summary [file 41467_2019_8538_MOESM4_ESM.pdf]

nature research

Corresponding author(s): DBPR: NCOMMS-18-19367B

Reporting Summary

Nature Research wishes to improve the reproducibility of the work that we publish. This form provides structure for consistency and transparency in reporting. For further information on Nature Research policies, see Authors & Referees and the Editorial Policy Checklist.

Statistical parameters

When statistical analyses are reported, confirm that the following items are present in the relevant location (e.g. figure legend, table legend, main text, or Methods section).

n/a	Confirmed	
☐	☒	The exact sample size (*n*) for each experimental group/condition, given as a discrete number and unit of measurement
☐	☒	An indication of whether measurements were taken from distinct samples or whether the same sample was measured repeatedly
☐	☒	The statistical test(s) used AND whether they are one- or two-sided<br>*Only common tests should be described solely by name; describe more complex techniques in the Methods section.*
☒	☐	A description of all covariates tested
☐	☒	A description of any assumptions or corrections, such as tests of normality and adjustment for multiple comparisons
☐	☒	A full description of the statistics including central tendency (e.g. means) or other basic estimates (e.g. regression coefficient) AND variation (e.g. standard deviation) or associated estimates of uncertainty (e.g. confidence intervals)
☐	☒	For null hypothesis testing, the test statistic (e.g. *F*, *t*, *r*) with confidence intervals, effect sizes, degrees of freedom and *P* value noted<br>*Give P values as exact values whenever suitable.*
☒	☐	For Bayesian analysis, information on the choice of priors and Markov chain Monte Carlo settings
☒	☐	For hierarchical and complex designs, identification of the appropriate level for tests and full reporting of outcomes
☒	☐	Estimates of effect sizes (e.g. Cohen's *d*, Pearson's *r*), indicating how they were calculated
☐	☒	Clearly defined error bars<br>*State explicitly what error bars represent (e.g. SD, SE, CI)*

Our web collection on statistics for biologists may be useful.

Software and code

Policy information about availability of computer code

Data collection	The Volocity version 6.3 was used to collect the intravital imaging data; the CytExpert software version 1.2 was used to collect the Flow cytometry data; the NIS-Elements software version 3.20 was used to collect the hematoxylin and eosin image data.
Data analysis	All fluorescence images were analyzed with Image J software version 1.48; Video was further edited with Adobe Premier Pro CC vesion 2017 1.1; The flow cytometry data were analyzed with FlowJo software version 10.7; Raw sequencing data were first filtered by Trimmomatic version 0.36; KEGG enrichment analysis of DEGs was implemented by KOBAS software version: 2.1.1; All statistical analyses were performed with GraphPad Prism software version 6.

For manuscripts utilizing custom algorithms or software that are central to the research but not yet described in published literature, software must be made available to editors/reviewers upon request. We strongly encourage code deposition in a community repository (e.g. GitHub). See the Nature Research guidelines for submitting code & software for further information.

Data

Policy information about availability of data

All manuscripts must include a data availability statement. This statement should provide the following information, where applicable:

- Accession codes, unique identifiers, or web links for publicly available datasets
- A list of figures that have associated raw data
- A description of any restrictions on data availability

Sequencing data have been deposited in Short Read Archive under project number PRJNA503822. The authors declare that all other data supporting the findings of this study are available within the article and its Supplementary Information files or from the corresponding authors on reasonable request

Field-specific reporting

Please select the best fit for your research. If you are not sure, read the appropriate sections before making your selection.

☒ Life sciences ☐ Behavioural & social sciences ☐ Ecological, evolutionary & environmental sciences

For a reference copy of the document with all sections, see nature.com/authors/policies/ReportingSummary-flat.pdf

Life sciences study design

All studies must disclose on these points even when the disclosure is negative.

Sample size	The required sample size was determined before the start of the study, corresponding number and tatistical methods were shown on the figure legends.
Data exclusions	No data were excluded from the analyses in the study.
Replication	The synthesis of nanoparticles should be controlled accurately (see, Methods,"Synthesis of nanoparticles" section) to verify the reproducibility of the experimental findings.
Randomization	To prevent selection bias, mice (6-8 weeks) were randomly allocated to different groups before treatment.
Blinding	The investigators were not blinded to group allocation during data collection and analysis.

Reporting for specific materials, systems and methods

Materials & experimental systems

n/a	Involved in the study	
☐	☒	Unique biological materials
☐	☒	Antibodies
☐	☒	Eukaryotic cell lines
☒	☐	Palaeontology
☐	☒	Animals and other organisms
☒	☐	Human research participants

Methods

n/a	Involved in the study	
☒	☐	ChIP-seq
☐	☒	Flow cytometry
☒	☐	MRI-based neuroimaging

Unique biological materials

Policy information about availability of materials

Obtaining unique materials	The authors confirm that all unique materials used are readily available from the standard commercial sources (see Method section)

Antibodies

Antibodies used	Methods,"Flow cytometry" section

Validation	The details of the antibody validation are stated on the manufacturer's websites (https://www.biolegend.com and https://www.thermofisher.com)

Eukaryotic cell lines

Policy information about cell lines

Cell line source(s)	Methods,"Mice and cells" section.
Authentication	Cell lines (B16F10, 4T-1, and CT26) were authenticated using short tandem repeat (STR) profiling.
Mycoplasma contamination	All cell lines were mycoplasma-negative as determined by screening using the MycoProbe Mycoplasma Detection Kit (R and D Systems, Minneapolis, MN, see Methods).
Commonly misidentified lines (See ICLAC register)	There are no commonly misidentified cell lines in the study.

Animals and other organisms

Policy information about studies involving animals; ARRIVE guidelines recommended for reporting animal research

Laboratory animals	Methods,"Mice and cells" section.
Wild animals	The study did not involve wild animals.
Field-collected samples	The study did not involve samples collected from the field.

Flow Cytometry

Plots

Confirm that:

☒ The axis labels state the marker and fluorochrome used (e.g. CD4-FITC).

☒ The axis scales are clearly visible. Include numbers along axes only for bottom left plot of group (a 'group' is an analysis of identical markers).

☒ All plots are contour plots with outliers or pseudocolor plots.

☒ A numerical value for number of cells or percentage (with statistics) is provided.

Methodology

Sample preparation	Methods, “Nonparenchymal liver cell preparation" and “Isolation of intrahepatic leukocytes” sections.
Instrument	CytoFLEX flow cytometer (Beckman Coulter, USA).
Software	The experiment data were collected by CytExpert software version 1.2 (Beckman Coulter, USA) and analyzed by FlowJo software (FlowJo, Ashland, OR, USA).
Cell population abundance	The abundance of relevant cell populations were described on relevant places (Page 5, 8, 10, and 11).
Gating strategy	The gating strategy to distinguish the hepatic myeloid cells was shown on Figure 1 legend. Conventional innate and adaptive immune components were gated by corresponding marks as shown in Supplementary Fig.2a-e

☒ Tick this box to confirm that a figure exemplifying the gating strategy is provided in the Supplementary Information.
